# Arsenic trioxide inhibits liver cancer stem cells and metastasis by targeting SRF/MCM7 complex

**DOI:** 10.1038/s41419-019-1676-0

**Published:** 2019-06-11

**Authors:** Hai-Yang Wang, Biao Zhang, Jun-Nian Zhou, Dong-Xing Wang, Ying-Chen Xu, Quan Zeng, Ya-Li Jia, Jia-Fei Xi, Xue Nan, Li-Juan He, Wen Yue, Xue-Tao Pei

**Affiliations:** 1 Stem Cell and Regenerative Medicine Lab, Institute of Health Service and Transfusion Medicine, Beijing, 100850 China; 2 South China Research Center for Stem Cell & Regenerative Medicine, SCIB, Guangzhou, 510005 China; 3 Experimental Hematology and Biochemistry Lab, Beijing Institute of Radiation Medicine, Beijing, 100850 China; 4 Department of Hepatobiliary Surgery, Beijing Tongren Hospital, Beijing, 100730 China

**Keywords:** Cancer stem cells, Metastasis

## Abstract

Hepatocellular carcinoma (HCC) has a high mortality rate due to the lack of effective treatments and drugs. Arsenic trioxide (ATO), which has been proved to successfully treat acute promyelocytic leukemia (APL), was recently reported to show therapeutic potential in solid tumors including HCC. However, its anticancer mechanisms in HCC still need further investigation. In this study, we demonstrated that ATO inhibits tumorigenesis and distant metastasis in mouse models, corresponding with a prolonged mice survival time. Also, ATO was found to significantly decrease the cancer stem cell (CSC)-associated traits. Minichromosome maintenance protein (MCM) 7 was further identified to be a potential target suppressed dramatically by ATO, of which protein expression is increased in patients and significantly correlated with tumor size, cellular differentiation, portal venous emboli, and poor patient survival. Moreover, MCM7 knockdown recapitulates the effects of ATO on CSCs and metastasis, while ectopic expression of MCM7 abolishes them. Mechanistically, our results suggested that ATO suppresses MCM7 transcription by targeting serum response factor (SRF)/MCM7 complex, which functions as an important transcriptional regulator modulating MCM7 expression. Taken together, our findings highlight the importance of ATO in the treatment of solid tumors. The identification of SRF/MCM7 complex as a target of ATO provides new insights into ATO’s mechanism, which may benefit the appropriate use of this agent in the treatment of HCC.

## Introduction

Hepatocellular carcinoma (HCC) is one of the most common solid tumors; more than 700,000 cases are diagnosed worldwide per year^1^. HCC has a high mortality rate due to the high metastatic potential and lack of effective treatments and drugs^2^. Thus, researchers have been making efforts to investigate more effective treatment options.

Arsenic trioxide (ATO), an FDA-approved drug as the first line treatment of acute promyelocytic leukemia (APL), is showing promising therapeutic potential in advanced liver cancers. As demonstrated by multiple clinical trials, HCC patients received ATO as a therapeutic agent or a chemosensitizer showed less extrahepatic metastasis and prolonged survival^3–5^. However, its anticancer mechanisms of action in HCC remain unclear. Therefore, clarifying the mechanism of ATO will help increase its clinical efficacy in HCC.

Previous researchers have reported that ATO induced growth inhibition and apoptosis in cancer cells^6,7^. Recently, several pieces of evidence also imply that ATO attenuates the cancer stem cells (CSCs) population with the involvement of SHH pathway^8,9^, Notch pathway^10,11^, and TGF-β pathway^12^. However, for most cancers, several types of inhibitors derived from normal stem cell-associated pathways such as SHH, Notch, and TGF-β have been proved to be ineffective in larger studies^13^. Therefore, the key functional molecules downstream of ATO which delineate its therapeutic effects in solid tumors including HCC need to be further identified.

Here, we demonstrated that ATO inhibits HCC lung metastasis and increases survival rate in a metastatic HCC xenograft model. Our results showed that ATO can inhibit the tumor-initiating capacity of HCC cells. To explore the underlying mechanism, target molecule alterations of HCC cells treated by ATO were investigated by a cDNA (complementary DNA) microarray. Minichromosome maintenance protein (MCM) 7 was further identified to be inhibited significantly after ATO treatment. Meanwhile, we found that MCM7 expression is correlated with HCC progression and prognosis in HCC patients. As we expected, MCM7 knockdown inhibits the tumor-initiating ability, and MCM7 ectopic expression abolishes the inhibitory effects of ATO. Most importantly, here for the first time, we report ATO downregulates MCM7 transcription by suppressing the transcription activity of serum response factor (SRF)/MCM7 complex. Taken together, we identify ATO as a new potential inhibitor of MCM7 protein in cancer, which will shed light on the treatment of MCM7-overexpressed HCC.

## Results

### ATO inhibits distant metastasis and prolongs survival of intrahepatic metastasis model mice

To minimize the toxicity of ATO on normal cells, we used various dosages of ATO to explore their effects on proliferation of normal human fetal liver L02 cells (Fig. S1A and B) and found that ATO at 3.6 μM or less had little effect on L02 cell apoptosis (Fig. S1C). Therefore, we used 3.6 μM for further assays in HCC cells and found that ATO at 3.6 μM had significant inhibitory effects on the proliferation (Fig. S1D), G0/G1 population (Fig. S1E), and the invasion of cells (Fig. S1F) but little effects on cell apoptosis (Fig. S1G). These results suggested that low dose of ATO inhibits HCC cell proliferation and invasion without inducing apoptosis.

**Fig. 1:**
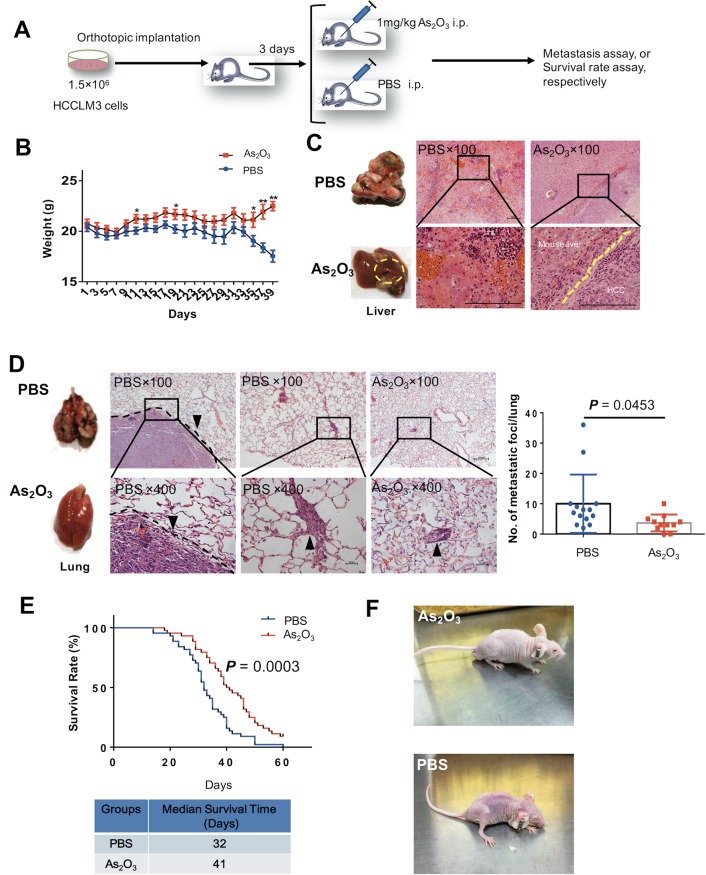
ATO inhibits HCC metastasis and prolongs survival in a mouse model. **a** A schematic diagram for the ATO therapy experiment. i.p. indicates intraperitoneal injection. **b**–**d** The lung metastasis assay. **b** The mice were weighted every other day (*n* = 14 for the PBS group, *n* = 11 for the ATO group). *P* values were determined by multiple *t*-tests. **c** Gross observation of nodules in livers (left) and representative images of H&E-stained liver sections (right) from ATO-treated mice and PBS-treated mice in the therapeutic experiments. Yellow circles indicate orthotopic tumors; magnification, bars, 100 μm. **d** shows representative examples of lungs (left) and representative images of H&E-stained lung sections (middle) from nude mice treated with ATO or PBS. Arrowheads indicate lung metastases foci. Bars, 100 μm in ×100, 20 μm in ×400. The different lung sections (5 sections per lung) were randomly chosen from each group, and the foci were quantified as the average number across the total sections per group (right). *P* = 0.0453, determined by two-sided Student’s *t*-test. **e** The survival curve of nude mice treated with ATO (*n* = 44) or PBS (*n* = 44) (top). *P* = 0.0003, by log-rank (Mantel–Cox) test. Bottom: the median survival time. **f** Appearance of the mice treated with ATO or PBS

Next, we investigated whether ATO inhibits distant metastasis in a metastatic HCC xenograft model, established as we previously reported^14–16^. The mice were given 1 mg/kg ATO (conversion from human dose) in H_3_AsO_3_ solution (As_2_O_3_ + 3H_2_O ↔ 2H_3_AsO_3_) or PBS by intraperitoneal injection every day and were examined for lung metastasis and survival (Fig. 1a). The weight of ATO-treated mice was significantly higher than that of control mice at the indicated day (Fig. 1b). The gross observations and hematoxylin and eosin (H&E) staining of tumor foci of the liver from both groups are shown in Fig. 1c. There were significantly fewer lung metastases in the ATO group than in the control group (*P* = 0.0453), in which tumors were visible on the lung surface (Fig. 1d). To study the long-term effects of ATO, we evaluated the survival rates between the two groups. The survival rate was significantly higher in the ATO group than the control group (median survival = 41 days and 32 days, respectively) (Fig. 1e). The experimental cancer cachexia was significantly improved in the surviving mice of the ATO group compared with the PBS group (Fig. 1f; Supplementary Video). Taken together, these results showed ATO can inhibit HCC metastasis and prolong survival in the mouse model.

### ATO inhibits liver CSCs-associated traits

**Fig. 2:**
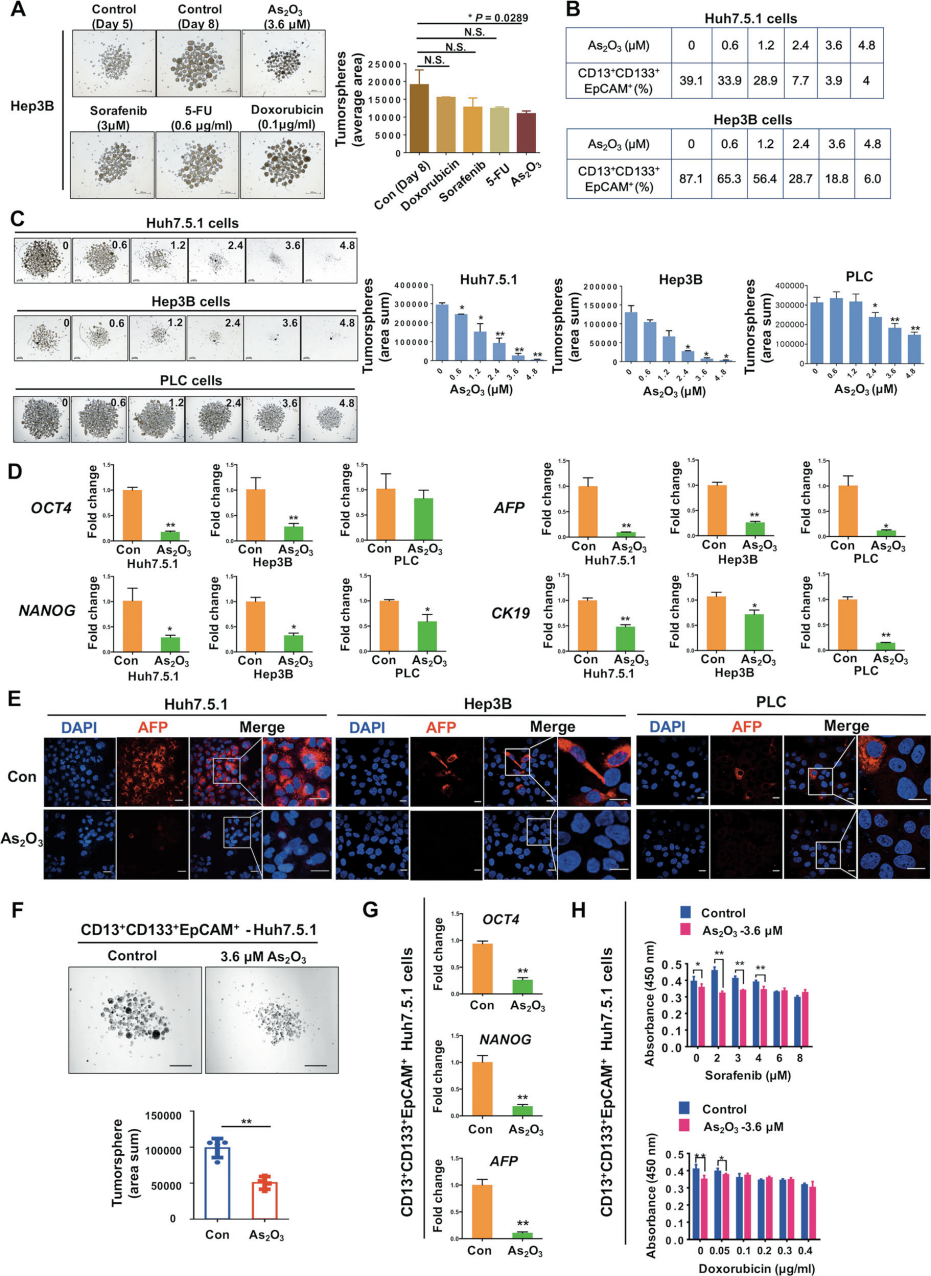
ATO attenuates liver CSC-associated traits in HCC cells. **a** The effects of ATO, sorafenib, 5-FU, and doxorubicin on tumorsphere formation. All the reagents were added into the tumorsphere system at day 5. Tumorsphere formation (*n* = 3) was quantified as the total cross-sectional areas of tumorspheres/number of tumorsphere per group (area average) at day 8. Bars, 500 μm. *P < 0.05, NS: not significant, determined by two-sided Student’s *t*-test. **b**, **c** The effects of ATO on CD13^+^/CD133^+^/EpCAM^+^ subpopulation percentage (**b**) and tumorsphere formation of HCC cells (**c**) at indicated concentrations (μM). Bars, 200 μm. Tumorsphere formation (*n* = 3) was quantified as the total cross-sectional areas of tumorspheres per group (area sum) (similarly hereinafter if not mentioned). “*” or “**” indicate the comparison for the “area sum” of tumorspheres with the control (0 μM) in the bar graphs. **d** Relative values of “stemness” gene and HCC marker gene expression in HCC cells. **e** Immunofluorescence analysis of AFP expressions after ATO treatment in HCC cells. Bars, 20 μm. **f**–**h** Comparison of tumorsphere formation (*n* = 3, **f**), relative values of “stemness” gene and HCC marker gene expression (by qRT-PCR, **g**), and chemoresistance (**h**) from Huh7.5.1 cells-derived CD13^+^/CD133^+^/EpCAM^+^ subpopulation treated with ATO or PBS. Bars, 500 μm. *P < 0.05, **P < 0.01, determined by two-sided Student’s *t*-test

Increasing evidence demonstrates that CSCs contribute to tumor initiation, distal metastasis, and chemotherapy resistance^17^. Thus, we next investigate whether ATO affects CSCs-associated characteristics in HCC cells. Tumorsphere, an effective in vitro model for analysis of the self-renewal capability of CSCs and enrichment of these cells from bulk cancer cells, were also assessed as we previously described^15,16^. We found that ATO could significantly decrease the tumorsphere formation compared with the control group, whereas doxorubicin, 5-FU, or sorafenib treatment did not show significant differences (Fig. 2a). Besides, we used a combination of reported liver CSC markers^18–20^ to enrich liver CSCs. We found that ATO decreased the CD13^+^CD133^+^EpCAM^+^ cell population (Fig. 2b and S2A and B) and tumorsphere formation (Fig. 2c) of HCC cells in a dose-dependent manner. Quantitative realtime polymerase chain reaction (qRT-PCR) analysis showed that the expression levels of stemness-associated genes, including *OCT4* and *NANOG*, and tumor markers, including *AFP* and *CK19*, in HCC cells were downregulated by ATO (Fig. 2d and S2C). Immunofluorescence analysis further showed that AFP expression was significantly downregulated (Fig. 2e) and the cell differentiation marker CK18 was abundant in cytoplasm after ATO treatment (Fig. S2D). To investigate whether ATO directly regulates the liver CSC population, we sorted the CD13^+^CD133^+^EpCAM^+^ cell population for further CSC assays. We found that the adherent growth (Fig. S2E), tumorsphere formation (Fig. 2f), expressions of *OCT4*, *NANOG*, and *AFP* (Fig. 2g), and chemoresistance capacity (Fig. 2h) of the sorted population were all inhibited by ATO. These results suggested that ATO inhibits liver CSCs-associated traits in vitro.

**Fig. 3:**
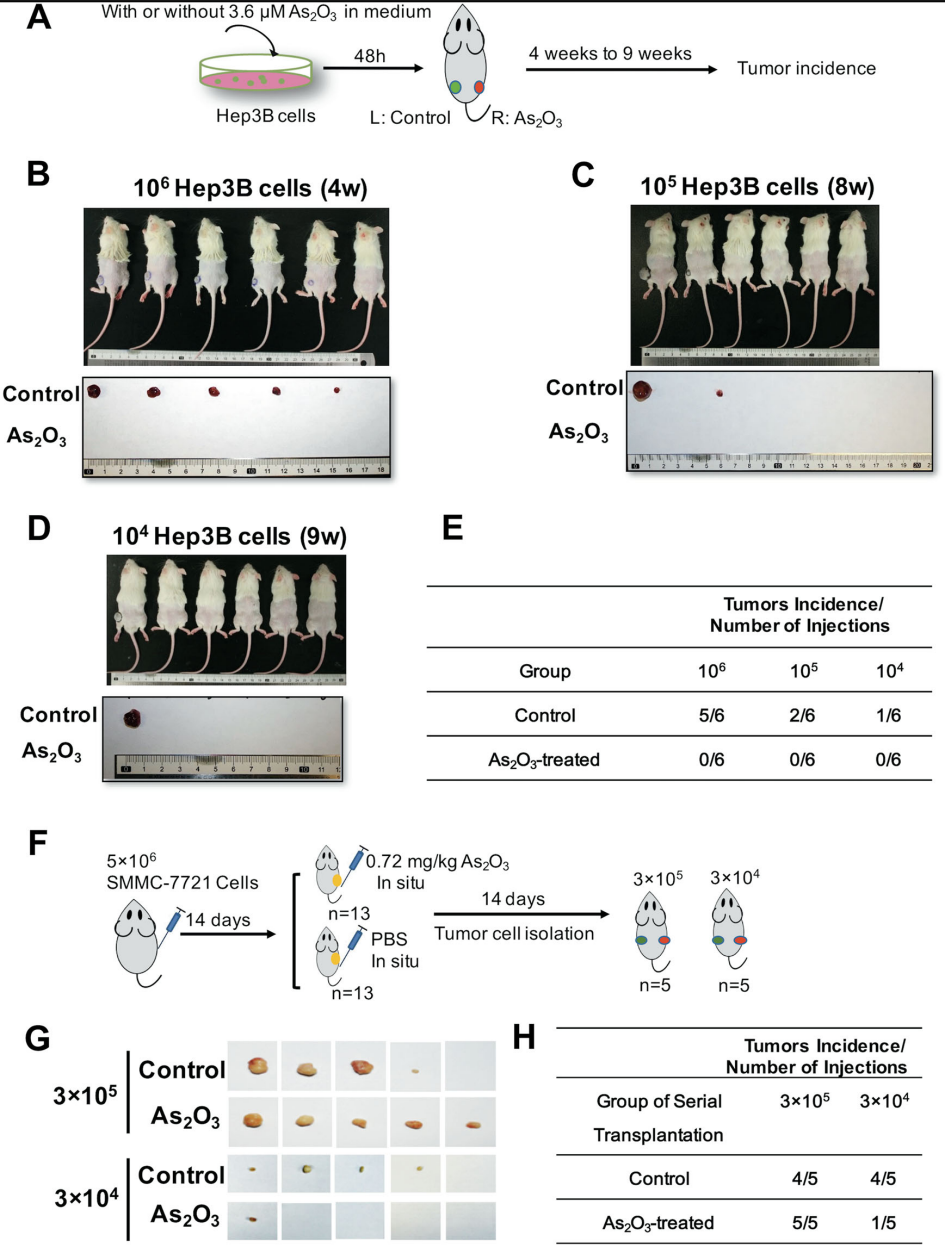
ATO inhibits HCC tumorigenic capacity. **a** A schematic diagram for examination of the effects of ATO on tumorigenic capacity of Hep3B cells. Subcutaneous injection of the Hep3B cells into NOD/SCID mice 48 h after HCC cells pre-treated with 3.6 μM ATO in 10% H-DMEM medium (HD). After 1 month, the peeled tumors were measured for size and weight. **b**–**d** Gross observation of tumor-bearing mice (top), peeled tumors (bottom), derived from Hep3B cells at 10^6^, 10^5^, and 10^4^ dilutions, respectively. **e** Tumor incidence of the cells from each group in diluting limitation assay. **f** A schematic diagram for examination of the effects of ATO on tumorigenic capacity of HCC cells via serial transplantation. ATO was given by local injection into the subcutaneous tumors of the nude mice every other day (100 μL) with the same volume of PBS as the control. After 2 weeks, the tumors were carefully removed and digested into single cells by the collagenase, which were then subcutaneously injected into the NOD/SCID mice. **g** Gross observation of the peeled tumors from each group. **h** Tumor incidence of the cells from each group in serial transplantation assay

To further investigate the effects of ATO on liver CSCs in vivo, we established an HCC xenograft model via subcutaneous injection of Hep3B cells (control group) or Hep3B cells treated with 3.6 μM ATO (ATO group) into the left or right flank of the same mouse, respectively (Fig. 3a). There were significant differences of tumor incidence in two groups (Fig. 3b–e). This effect of ATO on inhibiting tumor-initiation was also validated by using Huh7.5.1 cells (Fig. S3). We next performed serial transplantation of the SMMC-7721 cells derived from the subcutaneous tumors that received local injection of 0.72 mg/kg ATO in mice (Fig. 3f). Four mice in the PBS group (4/5) showed tumor growth, whereas only one mouse in the ATO group (1/5) showed growth at the 3 × 10^4^ dilution in serially transplanted mice (Fig. 3g, h), suggesting that ATO inhibits liver CSCs in vivo.

### MCM7 is downregulated in ATO-treated cancer cells

**Fig. 4:**
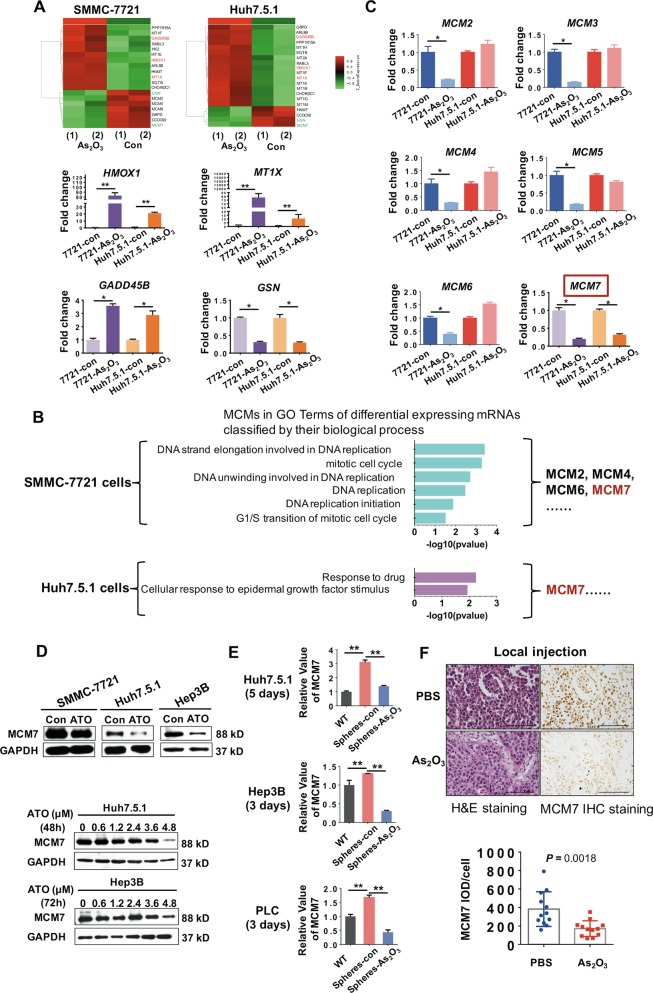
Investigation and confirmation of target molecule alterations by a cDNA microarray in ATO-treated HCC cells. **a** The microarray heat map (top) shows some of the profiles of differentially expressed mRNAs in two cells with over two-fold changes. Pseudo-colors indicate differential expression (green, transcript levels below the control; red, transcript levels greater than the control). Bottom: confirmation of some of the major candidate mRNAs by qRT-PCR analysis. *P < 0.05, **P < 0.01, determined by two-sided Student’s *t*-test. **b** GO analysis of differential expressing mRNAs classified by their biological process for MCM7 in HCC cells after ATO treatment. **c** qRT-PCR analysis of expressions of *MCM2*, *MCM3*, *MCM4*, *MCM5*, and *MCM6* in two cells after ATO treatment. *P < 0.05, **P < 0.01, determined by two-sided Student’s *t*-test. **d** Western blot (WB) analysis of MCM7 expression in HCC cell lines for 48 h of treatment with 3.6 μM ATO (top). Bottom shows MCM7 expression in HCC cell lines treated with ATO at the indicated dosages. **e** qRT-PCR analysis of *MCM7* expression levels in tumorspheres (*n* = 3) treated with ATO. WT, wild type adherent cells; Spheres-con, tumorspheres without ATO treatment; Spheres-ATO, tumorspheres with ATO treatment. *P < 0.05, **P < 0.01, determined by two-sided Student’s *t*-test. **f** H&E staining and MCM7 IHC staining of tumors derived from the mice that received local injection of ATO or PBS. Bars, 100 μm. **P < 0.01, *P < 0.05, determined by two-sided Student’s *t*-test

We then addressed the question of how ATO regulates liver cancer cells. We first investigated ATO-induced molecular alterations by using Affymetrix Human PrimeView mRNA microarray. A total of 201 genes were significantly changed in both ATO-treated HCC cell lines (fold change ≥2.0, *P* ≤ 0.05). Some of the candidate mRNAs that changed in both HCC cell lines, including *HMOX1*, *MT1X*, *GADD45B*, *GSN*, and *MCM7*, were validated by qRT-PCR analysis (Fig. 4a, c). Among these candidate mRNAs, MCM7, belonging to a highly conserved MCM family, attracted our attention. The MCM family, mainly consisted of MCM2, MCM3, MCM4, MCM5, MCM6, and MCM7, is essential for the initiation of eukaryotic genome replication^21^. Our gene ontology analysis of differentially expressed mRNAs classified by biological process demonstrated that *MCM7* is involved in cellular response to drug and proliferation stimulus, in addition to DNA replication and cell cycle^22^ (Fig. 4b). Moreover, among the MCM family members overexpressed in multiple cancers^23^, only *MCM7* was downregulated in both ATO-treated HCC cells (Fig. 4b, c). Therefore, we selected MCM7 for further investigation. RNA sequencing also showed that *MCM7* was overexpressed in multiple cancer tissues (Fig. S4). We next examined the expression level of MCM7 protein in ATO-treated HCC cells. ATO inhibited MCM7 expression in HCC cells in a dose-dependent manner (Fig. 4d). In tumorspheres, *MCM7* levels were upregulated compared with their adherent parental cells, while they were inhibited by ATO (Fig. 4e). Importantly, IHC staining of the tumors derived from mice that received local injection of ATO showed a significant reduction of MCM7 protein compared with control mice (Fig. 4f). These data suggested that ATO inhibits MCM7 expressions in vitro and in vivo.

### MCM7 is correlated with the tumor progression and prognosis in HCC patients

**Fig. 5:**
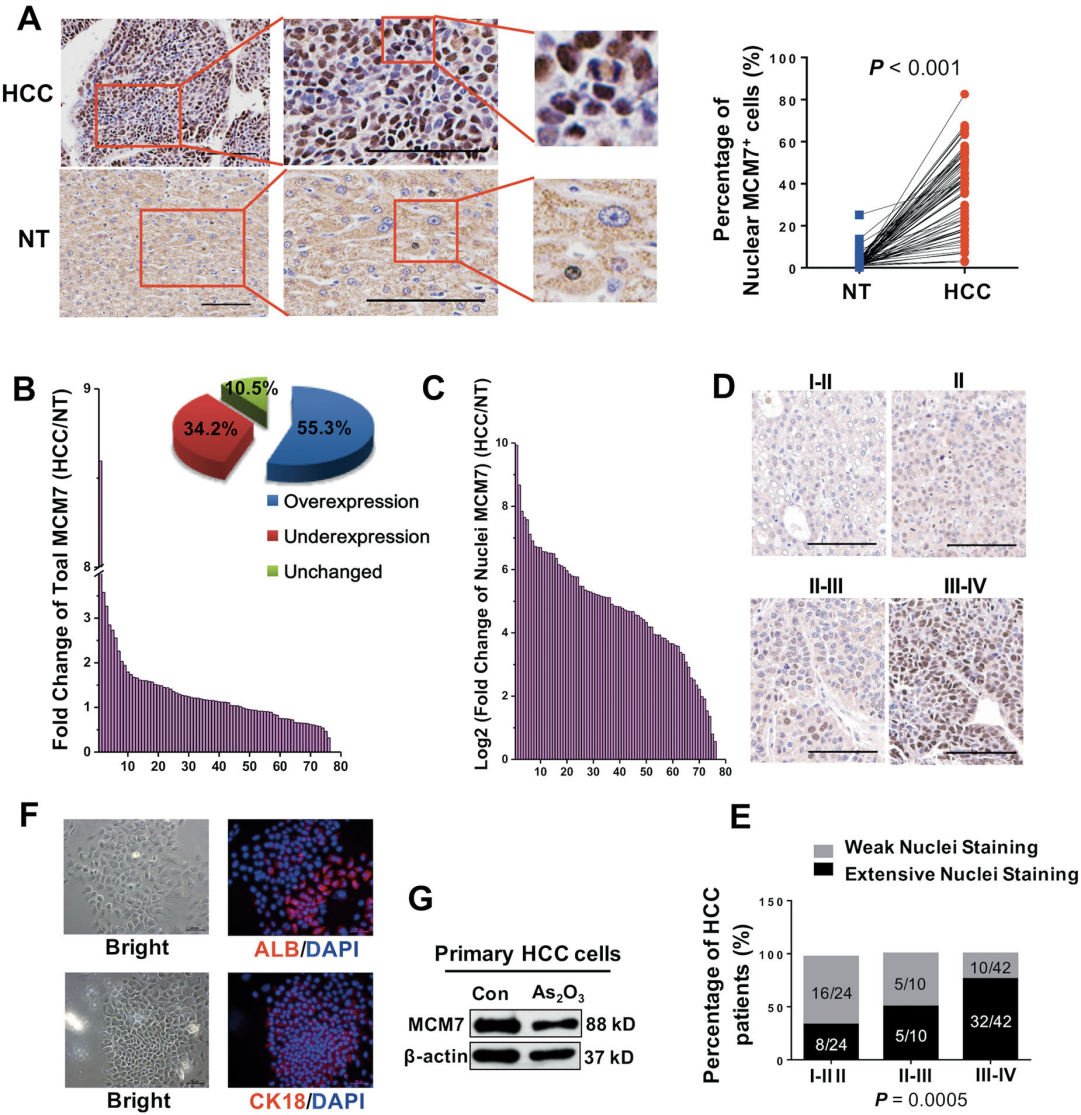
Correlation analysis of MCM7 and clinical grading in patients. **a** Representative examples of IHC staining of MCM7 (left) in HCC patients (*n* = 76). Quantitative analysis of the immunohistochemical expression of MCM7 (the number of MCM7 nuclear staining-positive cells/total cell number) in paired HCC tissues compared with the corresponding nontumor (NT) tissues (right). *P* < 0.0001, by paired *t*-test (two-sided). Bars, 100 μm. **b** A fold change (HCC/NT) plot (left) and a pie chart (right) of patient based on total MCM7 expression from HCC tissue arrays. **c** A log_2_ (HCC/NT) plot based on the ratio of nucleic MCM7 expression to total MCM7 expression. **d**, **e** Representative examples of IHC staining of MCM7 in HCC tissues at the indicated clinical grade (**d**). Bars, 100 μm. **e** The difference in percentage of MCM7 nuclear staining between the grade I-II/II, II-III, and III-IV groups of HCC patients (*P* *=* 0.0005, Fisher’s exact test). Weak nuclear staining: Integrated optical density (IOD) SUM of nuclear MCM7/IOD SUM of total MCM7 <30%; extensive nuclear staining: IOD SUM of nuclear MCM7/IOD SUM of total MCM7 >30%. **f** Immunofluorescence analysis of expression of ALB and CK18 in isolated primary HCC cells (left). Bars, 50 μm. **g** WB analysis of MCM7 expression in primary HCC cells treated with 3.6 μM of ATO

To clarify the clinical significance of MCM7, we investigated the expression profile of MCM7 in HCC tissues. We found an extensive nucleus staining of MCM7 in cancer tissues compared with a weak cytoplasmic staining in the paired nontumor tissues. A significant upregulation of MCM7 was observed in HCC tissues compared with the paired NT tissues (Fig. 5a, b). To exclude individual differences in MCM7 expression, we normalized the MCM7 expression intensities to those in the paired nontumor tissues in each pair. The total MCM7 expression was observed to be upregulated in 55.3% of HCC cases (Fig. 5b), whereas the MCM7 expression in the nuclei was strongly upregulated in 100% of HCC cases (Fig. 5c). Next, we performed a correlation analysis between the nucleic expression pattern of MCM7 and the histoprognostic factors. The elevated expression of MCM7 was highly correlated with the tumor diameter, Edmondson–Steiner grading, survival time, portal venous emboli, and AFP concentration of HCC patients (Fig. 5d, e and Supplementary Table 3). Moreover, we isolated human primary HCC cells (identified by epithelial makers including ALB and CK18, Fig. 5f). As we expected, a high MCM7 expression was observed and MCM7 expression was also inhibited by ATO treatment (Fig. 5g). Taken together, the elevated expression of MCM7 may play important roles in HCC progression.

### ATO inhibits liver CSCs and metastasis through targeting MCM7

**Fig. 6:**
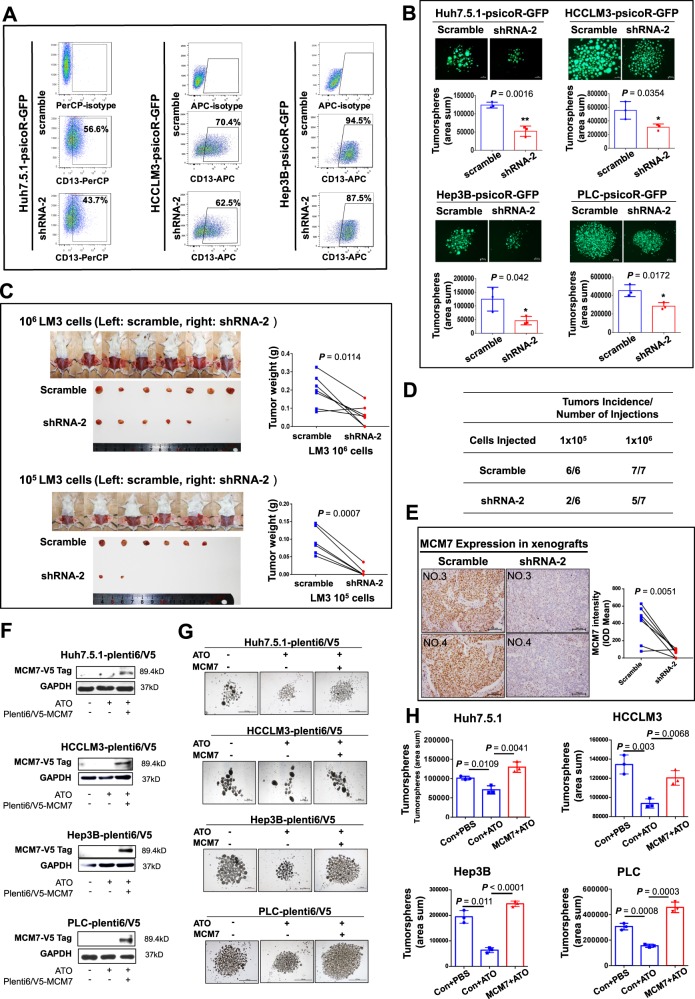
Analysis of MCM7 function in liver CSC and MCM7 “rescue” assays for ATO treatment. **a** The flow cytometry analysis of the CD13^+^ percentage in psicoR-shMCM7 stable cells. **b** shows a fluorescence image of the tumorspheres (*n* = 3) from psicoR-shMCM7-GFP cells and scramble cells. Bars, 200 μm. **c** Gross observation of tumor-bearing NOD/SCID mice and peeled tumors derived from HCCLM3-psicoR-shMCM7-GFP cells and scramble cells at two dilutions (left). Right shows the comparison of tumor weight between the shMCM7 group and control group. **d** The dilutions and mouse numbers. **e** Representative examples (left) and quantitative analysis (right) of the IHC staining of MCM7 in the tumors derived from HCCLM3-psicoR-shMCM7 cells and scramble cells. **f**–**h** Rescue assay of CSCs by the ectopic expression of MCM7. Western blot analysis of MCM7 expression in transfected HCC cells treated with ATO or not (**f**). Tumorsphere formation was observed (**g**) and quantified (**h**, *n* = 3). Bars, 1000 μm. *P* value was determined by two-sided Student’s *t*-test

To further answer the question whether ATO functions through MCM7 protein, we first validated the function of MCM7 in HCC cells. We established five stable MCM7-knockdown HCC cell lines (Fig. S5A and B), and found a decreased expression of the CSC marker CD13 (Fig. 6a). An increased expression of the cell differentiation marker CK18 is also observed in MCM7-knockdown cells (Fig. S6). Since MCM7 protein is upregulated in tumorspheres (Fig. S7), we performed tumorsphere forming assay and found that MCM7 knockdown significantly decreased the tumorsphere formation (Fig. 6b). Furthermore, we used psicoR-scramble/shMCM7 transfected Huh7.5.1 cells and HCCLM3 cells, the two cell lines with the most significant downregulation of MCM7 protein, to examine the tumor-initiating capacity of these HCC cells. The weight of tumors formed by HCCLM3-psicoR-shMCM7 cells was significantly decreased at both 1 × 10^6^ and 1 × 10^5^ dilutions, compared with scrambles (Fig. 6c). The tumor incidence was also decreased by MCM7 knockdown (Fig. 6d). For Huh7.5.1 cells, the scramble cells seeded at two dilutions had all formed tumors, whereas tumors were found in 7/7 and 5/7 mice in 1 × 10^6^ and 1 × 10^5^ knockdown group, respectively (Fig. S8A and B). The downregulation of MCM7 in formed tumors was confirmed by IHC staining (Figs. 6e and S8C). Moreover, MCM7 overexpression abolishes the inhibitory effects of ATO on tumorsphere formation (Fig. 6f–h). Our results demonstrated that downregulation of MCM7 recapitulates the inhibitory function of ATO on CSCs in vitro and in vivo.

**Fig. 7:**
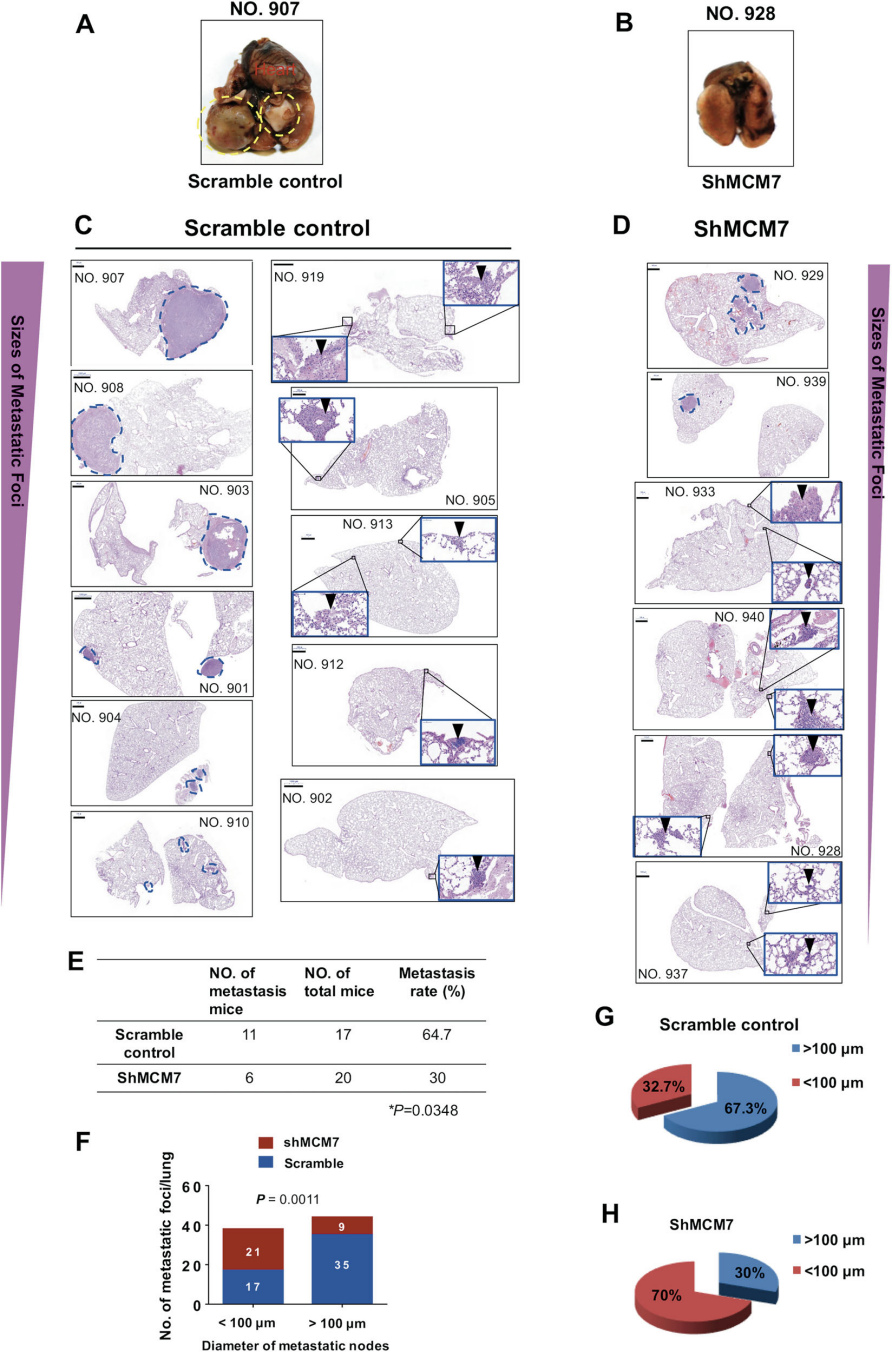
Effects of MCM7 knockdown on the metastasis capacity of HCC cells in vivo. **a–d** The nude mice received liver injections of MCM7-knockdown-HCCLM3 cells and scramble control cells, respectively. Representative images of gross observation of lungs (**a**, **b**) and lung metastasis foci in scanned panels of H&E-stained lung sections derived from both groups (**c**, **d**) were shown (Pannoramic MIDI, 3DHISTECH, Hungary). Blue dashed circles indicate typical macro-metastases foci (magnification: ×15) and arrowheads indicate typical micro-metastases foci in magnification inserts (×400). Bars, 1000 μm. **e** The metastasis rate of liver-inoculated mice with MCM7-knockdown HCC cells or control cells (*P* *=* 0.0348, by two-sided Chi-square test). **f** Quantitative analysis of the metastatic foci in detected lung sections based on the diameters (<100 μm and >100 μm) in each group. A total of 15 sections per lung (control group) and 17 sections per lung (MCM7-knockdown group) were randomly chosen and counted by two observers from each group. The loci diameters were measured by software (NIS-Elements F 4.30.01, Nikon). *P* = 0.0011, by two-sided Chi-square test. **g**, **h** A pie chart of the metastatic foci in detected lung sections based on the diameters in each group

We therefore examined the effects of MCM7 on metastasis in a metastatic HCC xenograft model^14–16^ (Fig. 7a–d). Inspiringly, MCM7 knockdown inhibited more than half of the distant metastases from primary tumors in liver to lung compared with the controls (30% vs. 64.7%, *P* = 0.0348) (Fig. 7e). It is reported that metastatic burden is a combination of both number of metastases and the size of the metastatic tumors^24^. Our further analysis showed that larger metastatic foci (>100 μm) were significantly reduced in MCM7-knockdown group in contrast to the control group (Fig. 7f–h). These results strongly supported that MCM7 downregulation has a significant inhibiting effect on the distant metastasis of HCC cells.

Collectively, our data revealed that ATO inhibits liver CSCs and the metastasis capacity of HCC cells through targeting MCM7.

### ATO downregulates MCM7 by suppressing the transcription activity of SRF

**Fig. 8:**
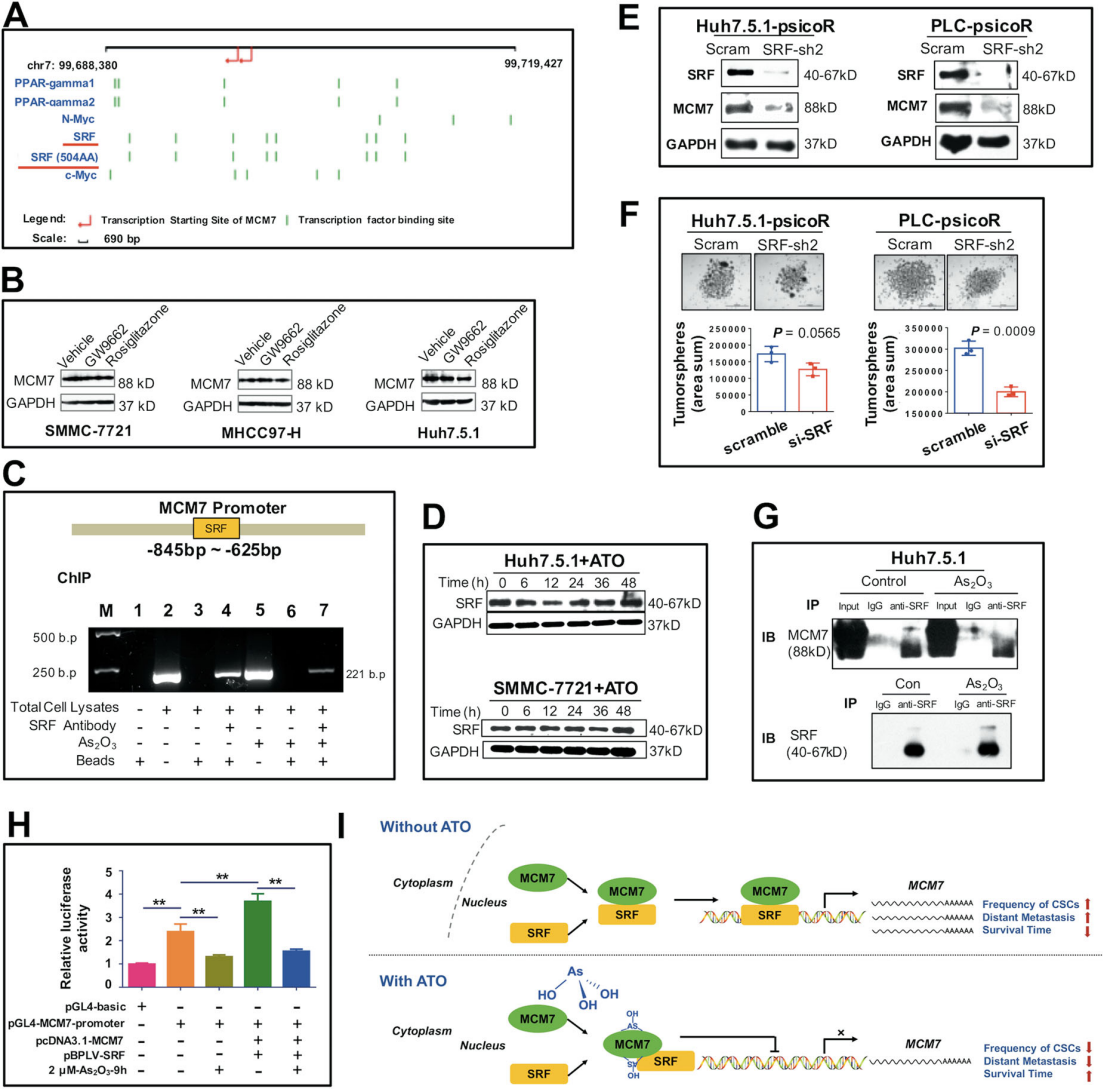
SRF-mediated regulation of *MCM7* transcription in ATO-treated HCC. **a** The transcription factor binding sites of *MCM7* promoter predicted by The Champion ChiP Transcription Factor Search Portal (http://www.sabiosciences.com/chipqpcrsearch.php?app=TFBS) based on SABiosciences’ proprietary database known as DECODE. **b** WB analysis of MCM7 expression in HCC cell lines 7 days after treatment with 10 μM of PPAR-γ antagonist GW 9662 (M6191, Sigma-Aldrich, St. Louis, MO) and 10 μM of PPAR-γ agonist Rosiglitazone (R2408, Sigma-Aldrich). **c** A ChIP assay for the detection of SRF and *MCM7* promoter binding specificity. M, DNA marker. **d** WB analysis of SRF expression at indicated time after ATO treatment in HCC cells. **e** WB analysis of SRF and MCM7 expression in SRF-knockdown HCC cells. **f** A brightfield image and quantification analysis of the tumorspheres from psicoR-shMCM7-GFP cells and scramble cells. Bars, 1000 μm. **g** IP with an anti-SRF antibody and analysis by immunoblotting with a MCM7 antibody and SRF antibody in HCC cells treated with ATO or PBS (con). **h** The dual luciferase assay for reporter gene expression in HeLa cells that were transfected with pGL4-MCM7-promoter luciferase constructs or a combination with pcDNA3.1-MCM7-expressing vectors plus pBPLV-SRF-expressing vectors, with a pGL4-basic vector as the control. Mean ± SD relative luciferase activity was calculated relative to Renilla activity. **P < 0.01, determined by Student’s *t*-test. **i** Summarized hypothesis on the ATO-SRF/MCM7 transcription axis in HCC. ATO interacts with MCM7 and inhibits the transcription activity of SRF/MCM7 complex, this in turn leads to the downregulation of MCM7, a key factor in HCC progression

To explore the regulating mechanisms of ATO on *MCM7* expression in HCC cells, we analyzed the possible transcription factors regulating *MCM7*. It is predicted that four transcription factors including peroxisome proliferator-activated receptor gamma (PPAR-γ), N-Myc, SRF, and c-Myc have the putative binding sites on *MCM7* promoter (Fig. 8a). MCM7 has been reported to be a direct target of the N-Myc in neuroblastoma^25^. We also used PPAR-γ antagonist GW 9662 and PPAR-γ agonist Rosiglitazone to test whether PPAR-γ regulates MCM7 expression but had negative results (Fig. 8b). SRF has been identified as an oncogenic driver of HCC that had elevated expression in HCC tissues, especially in high-grade, poorly differentiated tumors^26,27^, consistent with the expression pattern of MCM7 in HCC. Thus, we performed chromatin immunoprecipitation (ChIP) assays to test whether SRF bind to *MCM7* promoter in HCC cells. As shown in Fig. 8c, SRF binds to the *MCM7* promoter covering the region between −845 bp and −625 bp.

Interestingly, we found that ATO could inhibit the binding of SRF to *MCM7* promoter (Fig. 8c) but not constitutively downregulate the SRF expression (Fig. 8d). While in SRF-knockdown cells, MCM7 protein is significantly downregulated (Fig. 8e), the tumorsphere formation was also decreased (Fig. 8f). These results suggested that ATO downregulates MCM7 by suppressing the transcription activity of SRF in HCC cells.

### SRF/MCM7 transcription complex is involved in ATO inhibiting *MCM7* transcription

An interesting study suggested that MCM1 (a homolog of the mammalian SRF) in conjunction with MCM7 is involved in the regulation of *MCM7* expression in *Saccharomyces cerevisiae*^28^. We then addressed the question whether ATO disrupts the transcription activity of MCM7/SRF complex in HCC cells. We used immunoprecipitation (IP) assays with anti-SRF antibody and observed an MCM7 band, of which intensity was just slightly decreased in the precipitates obtained from the ATO-treated cells (Fig. 8g). Meanwhile, dual luciferase reporter assays showed that ATO can decrease the signal intensities of pGL4-*MCM7*-promoter. Transduction of pcDNA3.1-*MCM7* plus pBPLV-SRF resulted in an increase in *MCM7* transactivation, which can be significantly inhibited by ATO (Fig. 8h). These results indicated an MCM7/SRF complex also exists in HCC cells, through which ATO disrupt the transcription activity for *MCM7* without affecting the interaction between MCM7 and SRF.

Arsenic binds to various proteins containing cysteine residues^29^. Based on the distribution of cysteine (Cys, C) in the MCM7 protein and SRF protein, we speculated that arsenic might bind two adjacent cysteines of the MCM7 protein (Fig. S9A) by means of the As-OH, rather than the SRF protein with only one cysteine (Fig. S9B). Indeed, the dual immunofluorescence results indicated a colocalization for MCM7 and FlAsH-EDT2, a green biarsenical labeling reagent, in HCC cells (Fig. S9C). Furthermore, in vitro arsenic-protein binding assays demonstrated that FlAsH-EDT2 could bind to the full-length human recombinant MCM7 protein (Fig. S9D), while H_3_ASO_3_ significantly blocked the green fluorescence signals (Fig. S9E). We also examined whether ATO could directly degrade MCM7 protein. Even at 360 min after exposure, ATO did not induce a shift of MCM7 protein from the supernatant of cell lysates to the detergent-insoluble pellet separated from the RIPA buffer fraction as Zhang et al. previously reported^30^ (Fig. S9F). The enforced overexpression of MCM7 protein with pcDNA3.1-*MCM7-cmyc* plasmids was not reduced 48 h after ATO exposure (Fig. S9G). These results indicated that ATO may bind to the MCM7 protein but could not degrade the MCM7 protein directly.

From the data above, we supposed that ATO downregulates MCM7 expression possibly by inhibiting the transcription activity of SRF/MCM7 complex through binding MCM7 protein (Fig. 8i).

## Discussion

To date, CSC-targeting agents are rare or still undergoing clinical investigations. In this study, we offered a paradigm for identifying CSCs-targeted agents from approved drugs. To demonstrate the anti-CSCs potential of ATO, we provide compelling data obtained from in vitro tumorsphere formation assay, which is widely used in selection and enrichment of normal stem cells or CSCs^31^, and in vivo tumorigenic assay including serial transplantation analysis, which is recommended as the gold standard for the identification of CSCs^32,33^, and also results from a metastatic HCC xenograft model for estimating the metastatic potential of primary liver cancer. Moreover, we revealed MCM7 as a novel and direct target of ATO in HCC. This is the first study identifying direct downstream factors of ATO in liver CSCs by screening a total of 49,395 genes. The clinical significance of the target molecule in HCC specimens further provides solid evidence for the therapeutic value of ATO in clinical practice.

MCM7 has been shown a broad-spectrum overexpression in a variety of tumors including nervous system tumors^34,35^, prostate^36^, cervical^37^, epidermis^38^, and HCC^39,40^. Here, downregulation of MCM7 protein is demonstrated to be involved in ATO inhibiting liver CSCs. We found that HCC patients with elevated expression of MCM7 in tumor tissues are likely to have poor prognosis. The important roles of MCM7 in HCC progression further support the therapeutic significance of ATO, which dramatically inhibits MCM7 expression. Interestingly, in the last few years, MCM proteins including MCM7 have been utilized as diagnostic and prognostic tumor markers for the involvement of DNA replication process^41^. However, the abundance of MCM2–MCM7 proteins in cells is far more than the replication origins, suggesting that their functions may not be limited to DNA replication, this is termed as “MCM paradox”^21,22,42^. Here, our study provides a possible explanation for the “MCM paradox” by uncovering a stemness-associated regulatory function of MCM7 in ATO treatment.

There are lots of intensive investigations of the clinical effects and underlying mechanisms of ATO in leukemia^30,43,44^; however, solid tumors including HCC are distinct from leukemia in ontogeny patterns and molecular networks^6,45^. SRF is a ubiquitously expressed transcription factor regulating diverse genes involved in cell growth and differentiation, migration, and apoptosis^46^. Here, we uncovered the important role of SRF in ATO regulating MCM7 expression for the first time. We also found ATO binds two adjacent cysteines of the MCM7 protein rather than SRF by means of the As-OH, which is consistent with recent reports^29,47^. We suppose this binding might induce a conformational change in this SRF/MCM7 transcription complex and attenuate its binding to the promoter of *MCM7* (i.e., *MCM7* autoregulation) as previously reported^28^, which leads to the downregulation of *MCM7* transcription.

Our data suggest that ATO treatment in solid tumors, which has different regulatory mechanisms from those of standard chemotherapy, is of promising clinical applications. Given that only a few preclinical compounds inhibiting the MCM2–7 complex’s enzymatic activity and/or expression have been discovered^48^, and no FDA-approved drugs have been reported to target MCM complex, it is meaningful that we found ATO, approved in clinics, could target MCM7 in HCC.

Taken together, our data suggest that ATO is a promising therapeutic drug inhibiting liver CSCs and metastasis through MCM7 downregulation by suppressing the transcription activity of SRF/MCM7 complex. The identification of SRF/MCM7 complex as a direct target of ATO provides new insights into the drug’s mechanism of action, which may benefit the appropriate use of this agent in the treatment of solid tumors including HCC.

## Methods

### Cells and cell cultures

Human liver cancer cell lines (HCCLM3, Hep3B, Huh7.5.1, PLC, and SMMC-7721), the L02 human fetal liver cell line, and HEK-293T cell line were used. The HCCLM3 cells were from the Liver Cancer Institute of Fudan University (Shanghai, China). The Huh7.5.1 cells were maintained in our institute^16^. The Hep3B cells and SMMC-7721 cells were obtained from the Cell Bank of Shanghai Institute of Cell Biology, Chinese Academy of Sciences (Shanghai, China). PLC was obtained from ATCC. The HEK-293T packaging cell line was obtained from Invitrogen (Carlsbad, CA). The L02 cells were obtained from the National Platform for Experimental Cell Resources (Beijing, China). All cells were maintained in DMEM (Sigma) containing 10% FBS (ExCell Biology, Shanghai, China) at 37 °C with 5% CO_2_, except the L02 cells, which were maintained in RPMI 1640 medium (Sigma). Cell lines were subjected to routine cell line quality examinations (e.g., normal morphology, mycoplasma-free) and used less than 10 passages after thawed. The fresh primary liver cancer tissues were digested into single cells (Tumor Dissociation Kit, Miltenyi Biotec, Bergisch Gladbach, Germany) and cultured in the medium, kindly provided by Prof. Li-Jian Hui (Shanghai Institute of Biochemistry and Cell Biology, CAS)^49^. Fresh HCC tissues were provided from Beijing Tongren Hospital of China in accordance with the ethical standards of the institutional research committee and with the 1964 Helsinki Declaration and its later amendments or comparable ethical standards. Informed consent was obtained from the subjects. Tumor specimens were obtained according to protocols approved by the institutional review board of the Local Ethics Committee of Beijing, China.

### Tumorsphere culture

HCC cells were cultured in the tumorsphere system, as previously reported^15,16^.

### Chromatin immunoprecipitation (ChIP)

The HCC cells were treated by 3.6 μM ATO with PBS as the control. After incubation for 48 h, we cross-linked cells with 1% formaldehyde for 10 min at 37 °C and processed them in accordance with the Chromatin Immunoprecipitation Kit (#17-295, Millipore, Burlington, MA). Briefly, after cross-linking, the DNA was sheared, 4 μL of SRF-specific antibody (#5147S Cell Signaling Technologies, Danvers, MA) was used for immunoprecipitation overnight at 4 °C with rotation, followed by incubation with 60 μL of Protein G agarose for 1 h at 4 °C with rotation. Following the reverse cross-linking step, DNA was precipitated and purified by Axyprep DNA Gel Extraction Kit (AXYGEN, Corning, NY). The purified fragmented DNA was subjected for PCR amplification to detect relative occupancy. The specific primers used for human MCM7 promoter region with the SRF binding site are listed in Supplementary Table 1. The anti-SRF antibody used is listed in Supplementary Table 2.

### Animal models

Male Nu/Nu nude mice, aged 6–7 weeks, and male NOD/SCID mice, weight 18–20 g, were purchased from Vital River Laboratories (Beijing, China) or SPF (Beijing) Biotechnology Co., Ltd. (Beijing, China). Mice were housed and all experiments were performed according to international laws (EEC Council Directive 86/609, O.J. L 358. 1, December 12, 1987; Guide for the Care and Use of Laboratory Animals, United States National Research Council, 1996), relevant institutional guidelines and regulations of Beijing Medical Experimental Animal Care Commission. For diluting limitation assay of ATO-pretreated cells, 1 × 10^6^, 1 × 10^5^, and 1 × 10^4^ ATO-pretreated Hep3B cells, with the control cells, were subcutaneously injected into the flanks of NOD/SCID mice, respectively. The tumor growth was monitored weekly. The subcutaneous tumors were removed and weighed 4–9 weeks later. For subcutaneous tumorigenicity of MCM7-knockdown cells, HCCLM3-psicoR-shMCM7 cells and Huh7.5.1 psicoR-shMCM7 cells at two dilutions (1 × 10^6^ and 1 × 10^5^), with the control cells, were subcutaneously injected into the flanks of NOD/SCID mice, respectively. The subcutaneous tumors were removed and weighed 4 weeks later. For serial transplantation of ATO-treated tumors via local injection, 5 × 10^6^ SMMC-7721 cells were subcutaneously injected into the nude mice. Two weeks later, ATO at 0.72 mg/kg (in 100 μL) was locally injected into the subcutaneous tumors of the nude mice every other day with the same volume of PBS as the control. Approximately 2 weeks later, the tumors were carefully removed and were digested into single cells by Tumor Dissociation Kit (Miltenyi Biotec). The cells (two dilutions: 3 × 10^5^ and 3 × 10^4^) were subcutaneously injected into the left flank and the right flank of NOD/SCID mice (ATO-treated group vs. control group), respectively. The tumor growth was monitored weekly. The subcutaneous tumors were removed and weighed 8 weeks later. For intrahepatic metastasis model, 1.5 × 10^6^ cells (wild type of HCCLM3 cells followed by ATO treatment; MCM7-knockdown-HCCLM3 cells and scramble control cells) were suspended in 50 μL PBS containing 30% Matrigel for each nude mouse. Each mouse was orthotopically inoculated in the left hepatic lobe under anesthesia, through a 50-μL microinjector in the upper abdomen. If the mice died after injections of HCC cells into liver within 24 h, the mice would be excluded from the analysis. The mice were sacrificed 8 weeks later. For the self-controlled mice (i.e., NOD/SCID mice received subcutaneous injection of two group cells into the left flank and the right flank, respectively), The mice were randomly grouped. Otherwise, the mice were grouped by randomized block based on body weight.

### Statistical analysis

Two-sided Student’s *t*-tests, two-sided Chi-square test, Fisher’s exact test, and multiple *t*-tests were performed using GraphPad Prism 5.0 and Origin 6.1 software. Survival curves were analyzed and validated with the log-rank test. *P* values < 0.05 were considered significant. Results are shown as means ± S.D. The immunohistochemistry (IHC) staining intensity was analyzed using Image-Pro Plus 6.0 software (Media Cybernetics, Inc., Rockville, MD). The loci diameters were measured by software (NIS-Elements F 4.30.01, Nikon, Tokyo, Japan).

### Accession number

The microarray data have been submitted to the GEO database (http://www.ncbi.nlm.nih.gov/geo/) and assigned the identifier GSE111935.

## Supplementary information

Supplementary materials

Supplementary video
