# Supplementary material for: Arsenic trioxide inhibits liver cancer stem cells and metastasis by targeting SRF/MCM7 complex
Source: Cell Death Dis. 2019 Jun 11;10(6):453. doi: 10.1038/s41419-019-1676-0 (PMC6560089; doi:10.1038/s41419-019-1676-0)
Supplement: Supplementary file 1 — Supplementary materials [file 41419_2019_1676_MOESM1_ESM.pdf]

Supplementary Materials For

Arsenic Trioxide Inhibits Liver Cancer Stem Cells and Metastasis by Targeting SRF/MCM7 Complex

Hai-Yang Wang[1,3,*], Biao Zhang[1,3,*], Jun-Nian Zhou[1,2,3,#], Dong-Xing Wang[1,3], Ying-Chen Xu[4], Quan Zeng[1,3], Ya-Li Jia[1,2,3], Jia-Fei Xi[1,3], Xue Nan[1,3], Li-Juan He[1,3], Wen Yue[1,3,#], Xue-Tao Pei[1,3,#]

[#] Corresponding authors: zhoujunnian@alu.fudan.edu.cn (JNZ), yuewen@bmi.ac.cn (WY) and peixt@bmi.ac.cn (XTP)

This file includes:

Supplemental figures

Supplementary Figure 1: The biological effects of ATO on human normal fetal liver cell line L02 cells and HCC bulk cells.

Supplementary Figure 2: ATO attenuates liver CSC-associated traits in HCC cells.

Supplementary Figure 3: ATO inhibits tumorigenic capacity of Huh7.5.1 cells.

Supplementary Figure 4: MCM7 expression in multiple cancers tissues.

Supplementary Figure 5: Establishment of MCM7-knockdown HCC cells.

Supplementary Figure 6: Immunofluorescence analysis of CK18 expression in MCM7-knockdown HCC cells.

Supplementary Figure 7: Western blot analysis of MCM7 expression in tumorspheres.

Supplementary Figure 8: Effect of MCM7 knockdown on the tumorigenic capacity of Huh7.5.1 cells.

Supplementary Figure 9: ATO binds to MCM7 but does not directly degrade MCM7 protein.

Supplemental methods

Supplemental tables

Supplementary Table 1: The primers used in the study.

Supplementary Table 2: The antibodies used in the study.

Supplementary Table 3: The correlations between MCM7 nucleic expression pattern and clinico-pathologic factors in 76 HCC patients.

Supplemental references

Supplementary figures

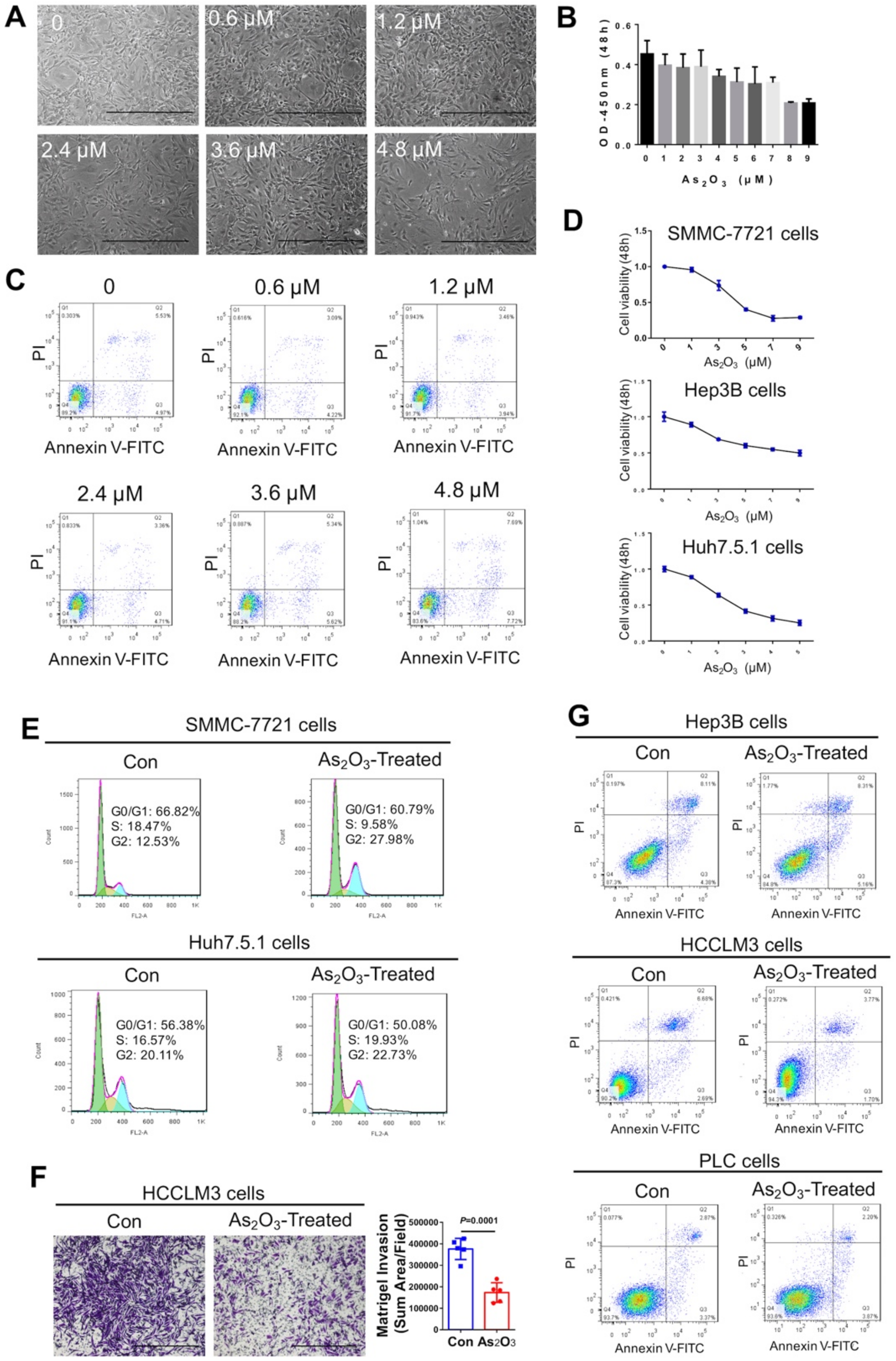

Supplementary Figure 1 The biological effects of ATO on human normal fetal liver cell line L02 cells and HCC bulk cells. (A) Morphological observation of L02 cells 48h after treatment with indicated concentrations of ATO. Bars, 200 μm. **(B)** Cell viability of L02 cells 48h after treatment with indicated concentrations of ATO. **(C)** Cell apoptosis assay of L02 cells 48h after treatment with indicated concentrations of ATO. **(D)** Cell viability of HCC cells 48h after treated with indicated concentrations of ATO. **(E)** Cell cycle analysis of HCC cells 48h after

treated with 3.6 μM of ATO. **(F)** Matrigel invasion assay of HCCLM3 cells, with a high invasive and metastasis capacity, 48h after treated with 3.6 μM of ATO. Bars, 500 μm. *P* value was determined by Student's t-test. **(G)** Cell apoptosis assay of HCC cells 48h after treated with 3.6 μM of ATO.

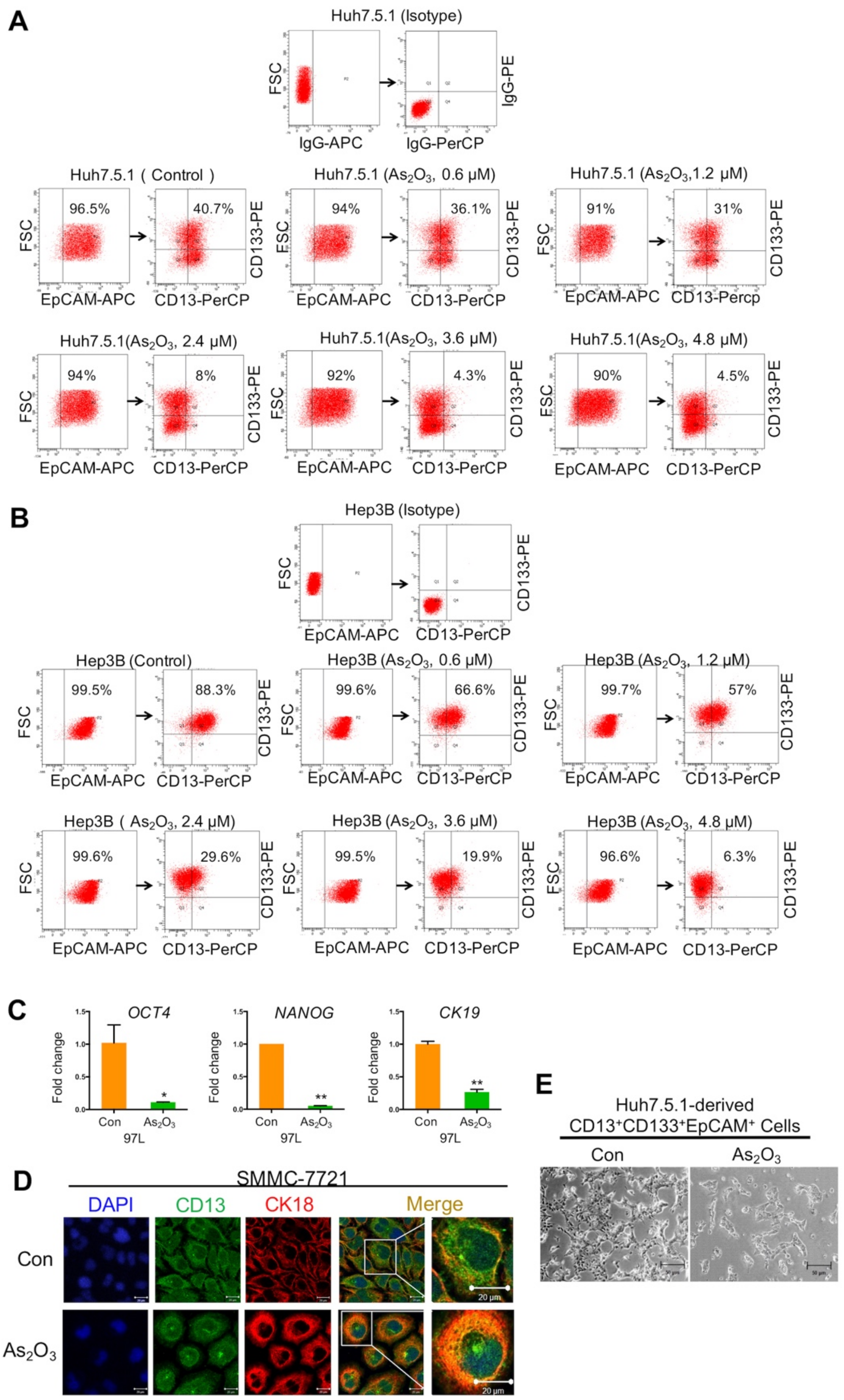

Supplementary Figure 2 ATO attenuates liver CSC-associated traits in HCC cells. (A, B) Flow cytometry scatter diagrams of CD13, CD133 and EpCAM (corresponding to Figure 2B) of Huh7.5.1 cells and Hep3B cells at indicated concentrations of ATO. **(C)** Relative values of *OCT4*, *NANOG* and *CK19* gene expression in 97L cells. **(D)** Immunofluorescence analysis of CD13 and CK18 expressions after ATO treatment in SMMC-7721 cells. Bars, 20 μm. **(E)** The appearance of the same number of Huh7.5.1-derived $CD13^{+}CD133^{+}EpCAM^{+}$ cells culture in 1:1 mixture of neurobasal medium (GIBCO) and DMEM/F12 medium (Life Technologies) supplemented with 0.5× N2 (GIBCO), 0.5× B27 supplements (GIBCO), 0.1% bovine serum albumin (AMRESCO), 2 mmol/L glutamine (R&D), 0.1 mmol/L 2-mercaptoethanol (Sigma, St. Louis, MO), 10 ng/mL BMP4 (R&D), 10 ng/mL basic fibroblast growth factor (bFGF, Peprotech, Rocky Hill, NJ), 10 ng/mL epidermal growth factor (EGF, R&D, Minneapolis, MN), 20 ng/mL hepatocyte growth factor (HGF, R&D), 20 ng/mL TGFα (Peprotech) (1), 48h after ATO treatment or not. Bars, 50 μm.

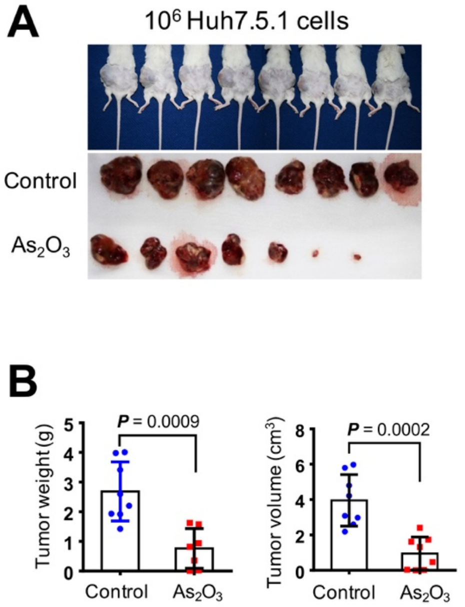

Supplementary Figure 3 ATO inhibits tumorigenic capacity of Huh7.5.1 cells. (A) Gross observation of tumor-bearing mice (top) and peeled tumors (bottom), derived from 10^{6} Huh7.5.1 cells. **(B)** Comparison of tumor weight and tumor size between ATO-treated group and control group.

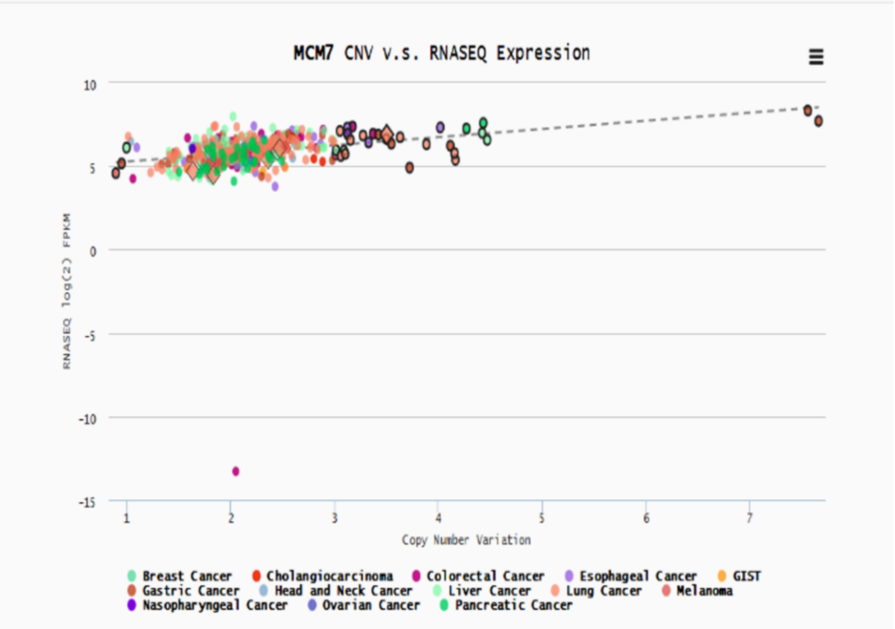

Supplementary Figure 4 MCM7 expression in multiple cancers tissues.
RNA sequencing data of MCM7 mRNA in multiple cancers tissues as indicated. (https://onco.wuxiapptec.com/search/gene).

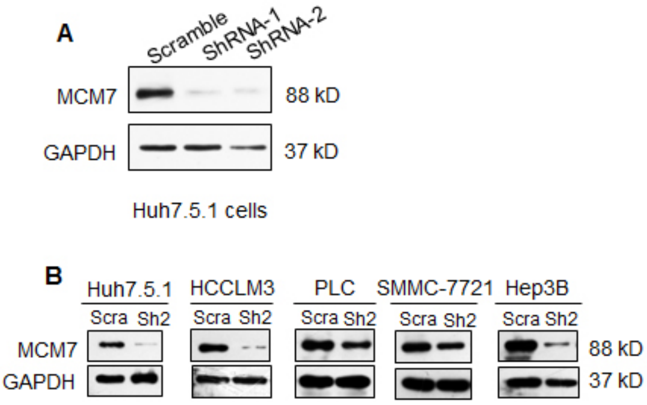

Supplementary Figure 5 Establishment of MCM7-knockdown HCC cells. (A) Validation the knockdown effects of two constructed psicoR-shMCM7-GFP lentivirus vector (shRNA-1 and shRNA-2) by western blot analysis in Huh7.5.1 cells with the scramble as controls (psicoR-scramble-GFP, scram). **(B)** Western blot analysis of MCM7 expression in HCC cells with stable knockdown of MCM7 by the psicoR lentivirus vector (psicoR-shMCM7) with the scramble as a control (psicoR-scramble).

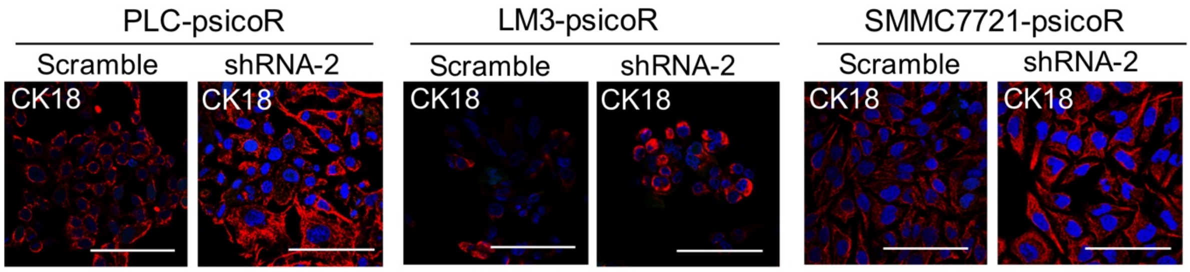

Supplementary Figure 6 Immunofluorescence analysis of CK18 expression in MCM7-knockdown HCC cells. CK18 expression of psicoR-shMCM7-GFP stable cells and control cells were evaluated under the same exposure conditions. Bars, 50 μm.

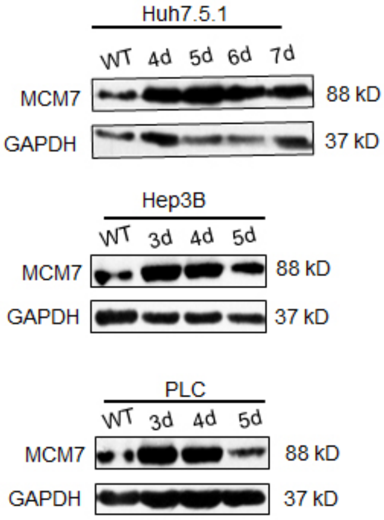

Supplementary Figure 7 Western blot analysis of MCM7 expression in tumorspheres. Western blot analysis of MCM7 expression in tumorspheres from three HCC cell lines at the indicated days. WT, wild type adherent cells.

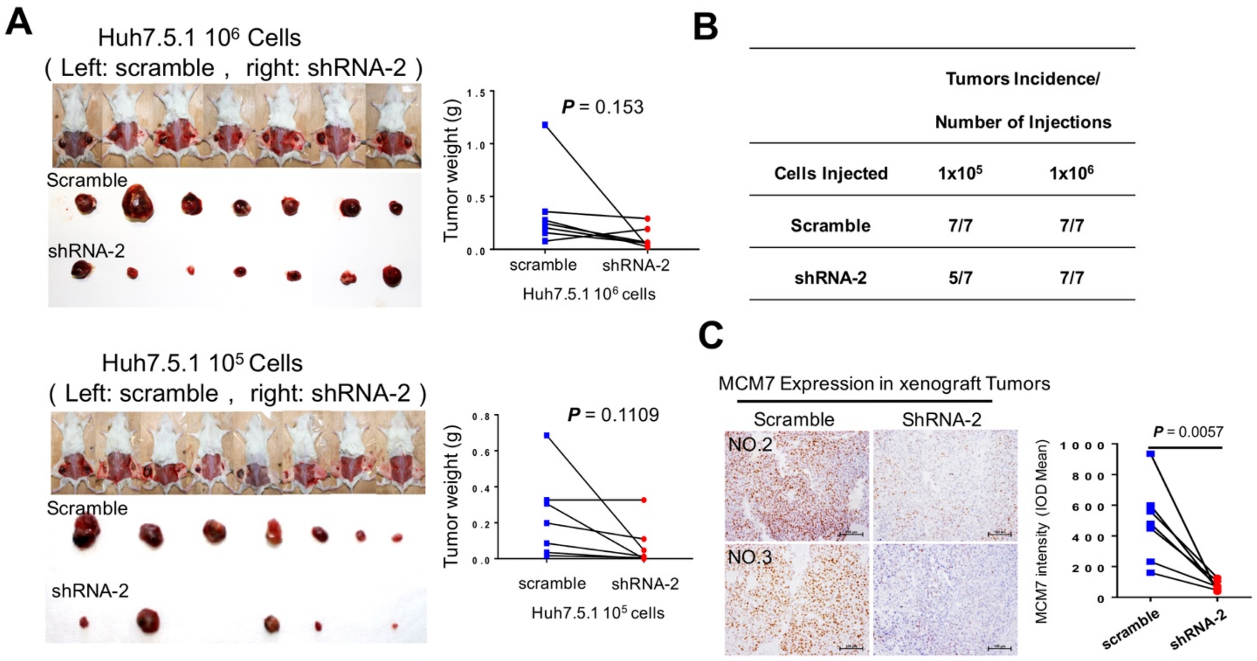

Cells Injected	Tumors Incidence/ Number of Injections $1x10^5$	$1x10^6$
Scramble	7/7	7/7
shRNA-2	5/7	7/7

Supplementary Figure 8 Effect of MCM7 knockdown on the tumorigenic capacity of Huh7.5.1 cells. (A) Gross observation of tumor-bearing NOD/SCID mice and peeled tumors derived from Huh7.5.1-psicoR-shMCM7-GFP cells and scramble cells at two dilutions (left). (Right) shows the comparison of tumor weight between the shMCM7 group and control group. **(B)** Tumor incidence of the cells from each group at each dilution. **(C)** Representative examples (left) and quantitative analysis (right) of the IHC staining of MCM7 in the tumors derived from Huh7.5.1-psicoR-shMCM7 cells and scramble cells.

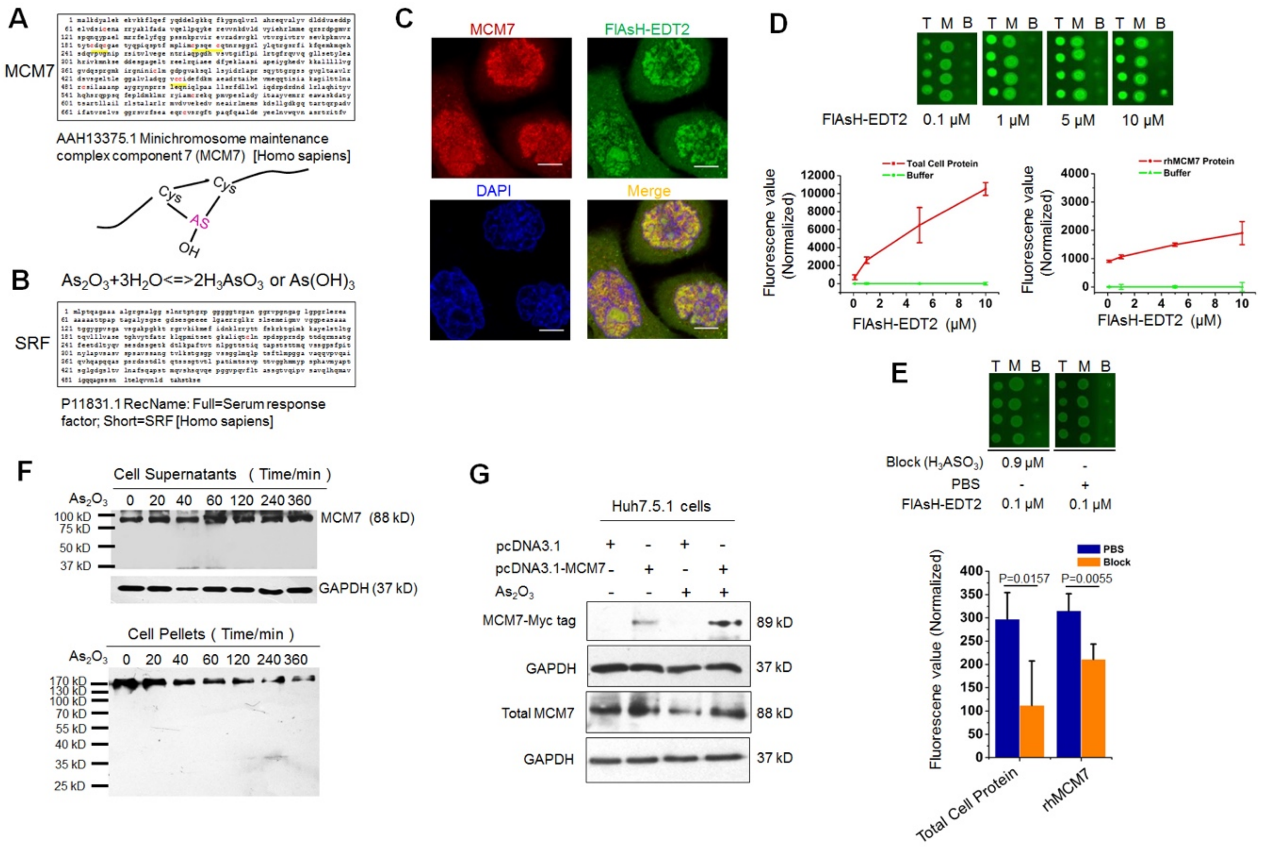

Supplementary Figure 9 ATO binds to MCM7 but does not directly degrade MCM7 protein. (A, B) A schematic diagram for arsenic binding to double Cys in the protein. The sequence of human MCM7 **(A)** and SRF **(B)** protein are listed. All the residues Cys (abbreviation, C) are marked with red colour in the sequence, respectively. The yellow lines highlight the double Cys with potential binding of arsenic in MCM7. **(C)** Dual immunofluorescence for MCM7 (red) and FlAsH-EDT2 (Green, a biarsenical labelling reagent) in HCC cells. Bars,10 μm. **(D, E)** *In vitro* arsenic-protein binding assay of coated purified MCM7 protein and FlAsH-EDT2 (green, a biarsenical labelling reagent) at the indicated concentrations **(D)** and a blocking experiment by H_3ASO_3 **(E)**. The green fluorescence signals were scanned by GenePix 4000B. T, total protein from 7721 cells lysed in RIPA buffer, as a positive control; M, recombinant human full-length MCM7 protein; B, Tris-HCl buffer as a negative control. **(F)** Western blot analysis of expressions of total MCM7 protein and MCM7-c-myc-tag fusion by anti-MCM7 antibody and anti-c-myc tag antibody, respectively, in Huh7.5.1-pcDNA3.1-MCM7 cells and Huh7.5.1-pcDNA3.1-con cells 48h after ATO treatment. **(G)** A time-course study showing the effects of ATO on MCM7 protein expression. Huh7.5.1 cells were treated with 3.6 μM ATO and then lysed in RIPA buffer and fractionated into supernatants (S) and pellets (P) by centrifugation at the indicated time.

Supplementary methods

Microarray

The microarray experiments were performed in the laboratory of the OE Biotech Company (Shanghai, China). For Huh7.5.1 cells and SMMC-7721 cells treated with ATO (Arsenious Acid and Sodium Chloride Injection, Heilongjiang Harbin Yida Pharmaceutical Co., Heilongjiang, China), the Affymetrix Human PrimeView Array was used for gene expression profiling analysis. Total RNA was quantified by the NanoDrop ND-2000 (ThermoFisher Scientific, Waltham, MA) and the RNA integrity was assessed using Agilent Bioanalyzer 2100 (Agilent Technologies, Santa Clara, CA). The sample labelling, microarray hybridization and washing were performed based on the manufacturer's standard protocols. Briefly, total RNA was transcribed to double strand cDNA, then synthesized cRNA and labelled with biotin. The labelled RNAs were hybridized onto the microarray. After washing, the arrays were scanned by the Affymetrix Scanner 3000 (Affymetrix, Santa Clara, CA). The array images were analysed with Affymetrix GeneChip Command Console (version 4.0, Affymetrix), followed by the basic analysis with the Genespring software (version 13.1; Agilent Technologies). The raw data was normalized with the RMA algorithm. Differential expression was determined based on fold change (FC) and the false discovery rate (FDR). The FDR was used to evaluate the significance of the *P*-value calculated with t-test. The threshold set for up-regulated and down-regulated mRNAs was a FC ≥ 2.0 and a $P \leq 0.05$. The data analysis including GO analysis and Kyoto Encyclopedia of Genes and Genomes (KEGG) analysis was performed by OE Biotech Company (Shanghai, China).

Vector constructs and cell transfections

The expression construct for human full-length MCM7 was generated by cloning PCR-amplified MCM7 cDNA fragments into the pcDNA3.1(-)/Myc-His vector (Invitrogen) for transient transfection. The pLenti6/V5-DEST-MCM7 (Addgene #31212) (2). was used for stable transfection following selection with blasticidin (Invitrogen, Carlsbad, CA; 5 μg/ml) for 5-7 days. The expression construct pBPLV-SRF-GFP was constructed by cloning PCR-amplified SRF cDNA from pCGN-SRF (addgene #11977). The promoter sequences of *MCM7* (1461 bp; Gene ID: 4176, position: -1461~0) were amplified via PCR using human genomic DNA and were inserted into the pGL4 basic vector (Promega, Madison, WI). To generate the plasmid for stable RNA interference (RNAi), hairpins were designed and cloned into psicoR-GFP lentiviral vector from Jacks Lab (MIT Center for Cancer Research, Cambridge, MA). A non-targeting, scrambled silencing RNA was used as control. The lentivirus packaging and cell transfection have been previously described (3-5). For transient transfections, 2-4 μg of plasmid DNA per six-well were used. The transfections were performed using Lipofectamine 2000 (Invitrogen, Carlsbad, CA) following the manufacturer's protocol. The cells were harvested for further assays 24-48 h after transfection. The total protein of the transfected cells was prepared 72 h after transfection.

Reverse-transcription PCR (RT-PCR) and quantitative RT-PCR (qRT-PCR)

RNA isolation, reverse transcription and qRT-PCR of genes were performed as previously described (3, 4). Total RNA was isolated using TRIzol reagent (Invitrogen) according to the manufacturer’s instructions. The experiments were performed with the ReverTra Ace qPCR RT Master Mix (TOYOBO, Osaka Prefecture, Japan) and the THUNDERBIRD SYBR qPCR Mix (TOYOBO) according to the manufacturer's protocol. The quantification of gene expression was based on

the △△Ct method and normalized to GAPDH RNA as an internal control. Quantitative analysis of mRNA levels was performed on the IQ5 Multicolor Real-Time PCR Detection System (Bio-Rad, San Diego, CA). All the primers are summarized in the supplementary Table 1.

Dual-luciferase reporter assay

The luciferase reporter assay was performed in triplicate. The 293T cells were co-transfected with 200 ng of pcDNA3.1-*MCM7* vector and/or 200 ng of pBPLV-*SRF* and 400 ng of pGL4-*MCM7*-promoter vector or pGL4 basic vector. The pRL *Renilla* luciferase vector (Promega, Madison, WI) was used to normalize the differences in transfection efficiency among the various groups. After transfection, the cells were treated with arsenic trioxide (3.6μM) or PBS (as controls) for 9 hours. The cells were lysed and then measured for luciferase activity using a Dual-Luciferase Reporter Assay System (Promega) 48 h after transfection according to the manufacturer's protocol.

In vitro arsenic-protein binding assay

The microarray slides were provided by CapitalBio Technology (Beijing, China). We used an Eppendorf pipette to absorb 1 μL prepared samples including total cell protein lysates (positive control), recombinant full length human MCM7 protein (Origene, Rockville, MD), and the Tris-HCl Buffer (negative control), and dropped them on the surface of the slides (n=4). Then the printed slides were placed on the drying oven and fixed overnight at room temperature. After washing with PBS and blocking with (or without) H_3AsO_3 solution, the FIAsH-EDT2 dye (Life Technologies Corporation) diluted in different concentrations were incubated with the array. After a

final washing, the arrays were scanned by GenePix 4000B scanner and analysed by GenePix Pro 6.0 software.

Immunoprecipitation (IP)

The HCC cells were treated by 3.6 μM ATO with PBS as the control. After incubation for 48 hours, cells were lysed in EBC lysis buffer (50 mM Tris-HCl, pH7.5; 100 mM NaCl, 1% NP-40) containing protease inhibitor cocktail (Roche, Basel, Switzerland). After 30 min on ice, the cell lysates were cleared by centrifugation at 4°C. The same appreciate amount of ATO-treated and control lysates were incubated with SRF-specific antibody (Cell Signaling Technologies, Danvers, MA) overnight at 4°C with rotation and Protein A/G PLUS-Agarose beads (Santa Cruz Biotechnology, Dallas, TX) for 1 h at 4°C with rotation. After three washes with EBC lysis buffer, the samples were boiled in loading buffer and immunoblotted with MCM7-specific antibody and SRF-specific antibody (Santa Cruz Biotechnology).

Cell viability assay

Cell viability was determined using the Cell Counting Kit-8 (CCK-8, Dojindo Laboratories, Kumamoto, Japan) as previously described (3). A total of 2×10^3-3×10^3 L02 cells or HCC cells per well were seeded in 96-well plates with DMEM (Sigma) containing 10% fetal bovine serum (FBS, Defined) (ExCell Biology, Shanghai, China). The cells were treated with ATO at the indicated concentration. The medium was exchanged for 100 μL DMEM plus 10 μL CCK-8 reagents and incubated at 37°C for 2 hours. Absorbance was measured at 450nm at indicated time. For doxorubicin and sorafenib chemoresistance assay, a total of 2×10^3-3×10^3 $CD13^+CD133^+EpCAM^+$ cells per well were seeded in 96-well plates. Twenty-four hours later, the medium was

replaced with doxorubicin (or sorafenib)-containing medium at the indicated concentration with or without 3.6 μM ATO and incubated for 48 h.

Cell apoptosis assay

The cell apoptosis was assayed with Annexin V, FITC Apoptosis Detection Kit (Dojindo Laboratories). The adherent L02 cells or HCC cells were pre-treated with ATO at the indicated concentration. After treatment for 48 hours, the cells were detached with EDTA-free trypsin and suspended at the concentration of 1×10^6 cells/ml in 1× Annexin V Binding solution. The cell suspension was incubated with 5 μL of FITC-Annexin V and 5 μL of PI Solution per 100 μL, followed by 15-minute incubation with protect from light. The apoptosis was analysed by flow cytometry analysis.

Matrigel invasion assay

HCCLM3 cells were pre-treated by 3.6 μM ATO with PBS as the control. After treatment for 48 hours, 2×10^6 HCCLM3 cells pre-treated with or without 3.6 μM ATO were added to the Matrigel (dilution 1:8, BD Biosciences, Franklin Lakes, NJ) - coated upper chamber of 6-well Hanging Cell Culture Inserts (8.0 μm pore size, Millipore, Billerica, MA) without FBS. The lower chamber was filled with the DMEM (Sigma Aldrich, St. Louis, MO) containing 10% FBS (ExCell Biology). After incubation for 72 hours, the cells in the bottom wells were fixed in formalin and stained with Crystal Violet (Sigma Aldrich). Each sample was repeated in 3 wells. Cells in 5 randomly selected fields (×100) in each well were counted.

Flow cytometry analysis and fluorescence-activated cell sorting (FACS)

The constructed stable cells and $CD13^+$ and $CD13^+/CD133^+EpCAM^+$ cells were isolated via sorting after transfection as previously described (3). The expression of

CD13, EpCAM and CD133 was examined via flow cytometry in the HCC cells. Briefly, the prepared HCC cells were washed twice with PBS and then incubated with PercP-conjugated anti-human CD13 (BD Pharmingen, Franklin Lakes, NJ) and/or APC-conjugated anti-human EpCAM (Biolegend, San Diego, CA), and/or PE-conjugated anti-human CD133 (Miltenyi Biotec, Bergisch Gladbach, Germany) for 30min at 4°C. PerCP-conjugated isotype (BD Pharmingen), APC-conjugated isotype (Miltenyi Biotec), PE-conjugated isotype (BD Pharmingen), were used as controls. After incubation, the cells were washed three times and then sorted on a FACS Aria instrument (BD Bioscience, Franklin Lakes, NJ). The expression of CD13, EpCAM and CD133 was examined via flow cytometry after ATO treatment or genetic manipulation in the HCC cells.

Immunofluorescence (IF) and immunohistochemistry (IHC) staining

Immunofluorescent staining and IHC staining were performed as previously described (3). Four-micrometer-thick paraffin sections were prepared using a Thermo Scientific Shandon Histocentre 3 Embedding Center (Thermo Scientific, Waltham, MA). Briefly, the fixed cells or tissue sections were incubated with primary antibodies overnight at 4°C after blocking, followed by incubation with fluorescein-conjugated secondary antibodies or HRP-conjugated secondary antibodies. MCM7 level in HCC tissues and corresponding nontumor (NT) tissues were evaluated by IHC on commercial tissue arrays (Super Biotek, Shanghai, China). The array contained 76 paired HCC and NT tissues. IHC staining was performed using PV kits (PV-6001 and PV-6002, Zhongshan Bio-tech Co., Ltd., Beijing, China) according to the manufacturer's instructions. Brightfield images of the H&E and IHC staining were collected with an ECLIPSE Ti microscope (Nikon). Confocal images were collected

by an LSM 510 META confocal system (Zeiss, Carl Zeiss MicroImaging, Jena, Germany).

The staining intensity of IHC was analysed using Image-Pro Plus 6.0 software (Media Cybernetics, Inc., Rockville, MD).

Western blots

Western blots were performed and analysed as previously described (3-5). Briefly, cells were lysed in RIPA buffer (50 mM Tris-HCl, pH 7.5, 150 mM NaCl, 1 % NP-40, 0.5 % sodium deoxycholate, 0.1% SDS) supplemented with protease inhibitors (Roche) overnight at 4°C with rotation. The whole-cell lysates were fractionated into supernatants and pellets by centrifugation. The cell lysates were separated on SDS-PAGE and transferred to PVDF membranes (Millipore). The blots were blocked with TBST containing 5 % skimmed milk and incubated with primary antibodies overnight at 4°C, followed by incubation with HRP-conjugated secondary antibodies (Zhongshan Bio-tech Co., Ltd.) for 1 h. Immunoreactivity was detected and the signals were analysed were detected via Immobilon Western Chemiluminescent HRP Substrate (Millipore). All the antibodies used in this study are summarized in the supplementary Table 2.

Supplemental tables

Supplementary Table 1 The primers used in the study.

**Gene**	**Forward primer (5'-3')**	**Reverse primer (5'-3')**
AFP	CTTTGGGCTGCTCGCTATGA	GCATGTTGATTTAACAAGCTGCT
CK19	ACCAAGTTTGAGACGGAACAG	CCCTCAGCGTACTGATTTCCT
OCT-4	AGCAAAACCCGGAGGAGT	CCACATCGGCCTGTGTATATC
NANOG	TCCCGAGAAAAGATTAGTCAGCA	AGTGGGGCACCTGTTTAACTT
GSN	AGATGGACTACCCCAAGCAGA	GGTCCCGCCAGTTCTTGAA
MT1X	GACCCCAACTGCTCCTGCTCG	GATGTAGCAAACGGGTCAGGGTTGTAC
HMOX1	AAGACTGCGTTCCTGCTCAAC	AAAGCCCTACAGCAACTGTCG
GADD45B	TACGAGTCGGCCAAGTTGATG	GGATGAGCGTGAAGTGGATTT
MCM2	CACCACCGCTTCAAGAACTTCC	TTCACCACCAGGCTCTCACG
MCM3	CCAAGCAGTATGAGGAGTTC	CTTCTTAGTAGCAGGACAGTAG
MCM4	GACGTAGAGGCGAGGATTCC	GCTGGGAGTGCCGTATGTC
MCM5	AGCATTCGTAGCCTGAAGTCG	CGCATGGCAATGTTGGTGAG
MCM6	CAGTGGTGTTGATGGATATG	CATTCTTTCACAGTCATTTGG
MCM7	CCTACCAGCCGATCCAGTCT	CCTCCTGAGCGGTTGGTTT
GAPDH	GAGTCAACGGATTTGGTCGT	TTGATTTTGGAGGGATCTCG
MCM7 CDS cloning primers	GCTCTAGAGCGCCACCATGGCACTGAAGGACTAC	GGGGTACCGACAAAAGTGATCCGTGTCCGGGA
SRF CDS cloning primers	TCTAGAATGTTACCGACCCAAGCT	GTCGACTCATTCACTCTTGGTGCTGT

MCM7 promoter cloning primers	GCTAGCTAAAGGAAGGACAGTCTGAGTGATCA	CTCGAGGCCAGGAGACGAGTCCCCGGTT
MCM7 promoter primers for CHIP	TCACCTGGAGAGCAGTTCTAGGA	TGCTGTTGTGACTAGTAAGA
MCM7 promoter primers for CHIP	TCACTGCAACCTCTGCCTCTT	ACTGGTTCATTGGTAGATT
MCM7 shRNA-1 primers	TggaaatatccctcgtagtaTTCAAGAGAtactacgagggatatttccTTTTTTC	TCGAGAAAAAAggaaatatccctcgtagtaTCTCTTGAAtactacgagggatatttccA
MCM7 shRNA-2 primers	TggctaatggagatgtcaaaTTCAAGAGAtttgacatctccattagccTTTTTTC	TCGAGAAAAAAggctaatggagatgtcaaaTCTCTTGAAtttgacatctccattagccA
SRF shRNA-1 primers	TcgatgtttgccatgagtattaTTCAAGAGAtaatactcatggcaaacatcgTTTTTTC	TCGAGAAAAAAcgatgtttgccatgagtattaTCTCTTGAAtaatactcatggcaaacatcgA
SRF shRNA-2 primers	TgtgagacaggccatgtgtataTTCAAGAGAtatacacatggcctgtctcacTTTTTTC	TCGAGAAAAAAgtgagacaggccatgtgtataTCTCTTGAAtatacacatggcctgtctcacA
shRNA-scramble primers	TTTCTCCGAACGTGTCACGTTTCAAGAGAACGTGACACGTTCGGAGAATTTTTTC	TCGAGAAAAAATTCTCCGAACGTGTCACGTTCTCTTGAAACGTGACACGTTCGGAGAAA

Supplementary Table 2 The antibodies used in the study.

**Protein**	**Applications**	**Antibody**	**Origin**	**Dilution**	**Molecular weight/Localization**
APC-CD13	FC	130-103-734, Miltenyi Biotec	mouse	5 μL/test	Cell membrane
APC-IgG Isotype	FC	130-104-614, Miltenyi Biotec	mouse	5 μL/test	
APC-EpCAM	FC	324208, Biolegend	mouse	5 μL/test	Cell membrane
APC-IgG Isotype	FC	400327, Biolegend	mouse	5 μL/test	
PerCP-Cy™5.5-CD13	FC	561361, BD Pharmingen™	mouse	5 μL/test	Cell membrane
PerCP-Cy™5.5-IgG Isotype	FC	550795, BD Pharmingen™	mouse	5 μL/test	
PE-CD133	FC	130-080-801, Miltenyi Biotec	mouse	5 μL/test	Cell membrane
PE-IgG Isotype	FC	555574, BD Pharmingen™	mouse	10 μL/test	
CD13	IHC	sc-166270, Santa Cruz Biotechnology	mouse	1:200	Cell membrane/ cytoplasmic
CD13	IF	ab7417, Abcam	mouse	1:200	Cell membrane/ cytoplasmic
AFP	IF	NB100-1611, Novus Biologicals	rabbit	1:200	Cytoplasmic
MCM7	WB, IHC	sc-9966, Santa Cruz Biotechnology	mouse	1:1000, 1:200	88 kDa/Nuclei, cytoplasmic
MCM7	IF	3735S, CST	rabbit	1:200	Nucleus/cytoplasmic
SRF	IF, CHIP, IP	5147S, CST	rabbit	1:200, 1:500, 1:100	Nucleus
SRF	WB	sc-335x, Santa Cruz Biotechnology	rabbit	1:1000	67kDa/Nucleus
CK18	IF	ab133263, Abcam	rabbit	1:200	Cytoplasmic
CK19	IHC	ab52625, Abcam	rabbit	1:200	Cytoplasmic
MYC-Tag	WB	2276S, CST	mouse	1:1000	
V5-Tag-HRP	WB	PM003-7, MBL	rabbit	1:5000	
GAPDH-HRP	WB	8884, CST	rabbit	1:1000	Cytoplasmic

Supplementary Table 3 The correlations between MCM7 nucleic expression pattern and clinico-pathologic factors in 76 HCC patients

		MCM7 Nuclei Staining[1]		
		**≤ 30% (No. of cases)**	**>30% (No. of cases)**	**P-value**
Tumor size	≤ 8 cm	26	25	0.0054**
	> 8 cm	4	21	
Cellular differentiation by Edmondson-Steiner grading	I-II & II	16	8	0.0005**
	II-III	5	5	
	III & III-IV	10	32	
Survival time	≤500 days	9	27	0.0191*
	>500 days	21	19	
Portal venous emboli	Positive	2	14	0.0195*
	Negative	28	32	
AFP (ng/mL)	≤300	21	20	0.0339*
	>300	9	26	
Distant metastasis	Positive	2	3	1.000
	Negative	28	43	
HBV infection	Positive	26	41	0.7331
	Negative	4	5	
Gender	Male	28	37	0.1837
	Female	2	9	

[1] Integrated optical density (IOD) SUM of nuclei MCM7/IOD SUM of total MCM7

*$P < 0.05$, **$P < 0.01$ determined by Fisher's exact test.

Supplementary references

1. **Zhang L, Sun H,** Zhao F, Lu P, Ge C, Li H, Hou H, et al. BMP4 administration induces differentiation of CD133+ hepatic cancer stem cells, blocking their contributions to hepatocellular carcinoma. Cancer Res 2012;72:4276-4285.
2. **Scott KL, Nogueira C, Heffernan TP**, van Doorn R, Dhakal S, Hanna JA, Min C, et al. Proinvasion metastasis drivers in early-stage melanoma are oncogenes. Cancer Cell 2011;20:92-103.
3. **Zhou JN, Zeng Q,** Wang HY, Zhang B, Li ST, Nan X, Cao N, et al. MicroRNA-125b attenuates epithelial-mesenchymal transitions and targets stem-like liver cancer cells through small mothers against decapentaplegic 2 and 4. Hepatology 2015;62:801-815.
4. **Yan XL, Jia YL,** Chen L, Zeng Q, Zhou JN, Fu CJ, Chen HX, et al. Hepatocellular carcinoma-associated mesenchymal stem cells promote hepatocarcinoma progression: role of the S100A4-miR155-SOCS1-MMP9 axis. Hepatology 2013;57:2274-2286.
5. **Jia YL, Shi L,** Zhou JN, Fu CJ, Chen L, Yuan HF, Wang YF, et al. Epimorphin promotes human hepatocellular carcinoma invasion and metastasis through activation of focal adhesion kinase/extracellular signal-regulated kinase/matrix metalloproteinase-9 axis. Hepatology 2011;54:1808-1818.
